# On the Structure, Stability, and Cell Uptake of Nanostructured Lipid Carriers for Drug Delivery

**DOI:** 10.1021/acs.molpharmaceut.4c00392

**Published:** 2024-06-05

**Authors:** Ramona Jeitler, Christina Glader, Gerhard König, Jay Kaplan, Carolin Tetyczka, Johan Remmelgas, Marion Mußbacher, Eleonore Fröhlich, Eva Roblegg

**Affiliations:** † Institute of Pharmaceutical Sciences, Pharmaceutical Technology and Biopharmacy, University of Graz, 8010 Graz, Austria; ‡ Research Center Pharmaceutical Engineering GmbH, 8010 Graz, Austria; § Centre for Enzyme Innovation, School of Biological Sciences, University of Portsmouth, Portsmouth PO1 2DY, United Kingdom; ∥ Pritzker School of Molecular Engineering, University of Chicago, Chicago, Illinois 60637, United States; ⊥ Institute of Pharmaceutical Sciences, Pharmacology and Toxicology, University of Graz, 8010 Graz, Austria; # Center for Medical Research, Medical University of Graz, 8010 Graz, Austria

**Keywords:** nanostructured lipid carriers, nanocarrier structural organization, molecular interactions, physical stability, cellular interactions and uptake, coarse-grained molecular dynamics simulations

## Abstract

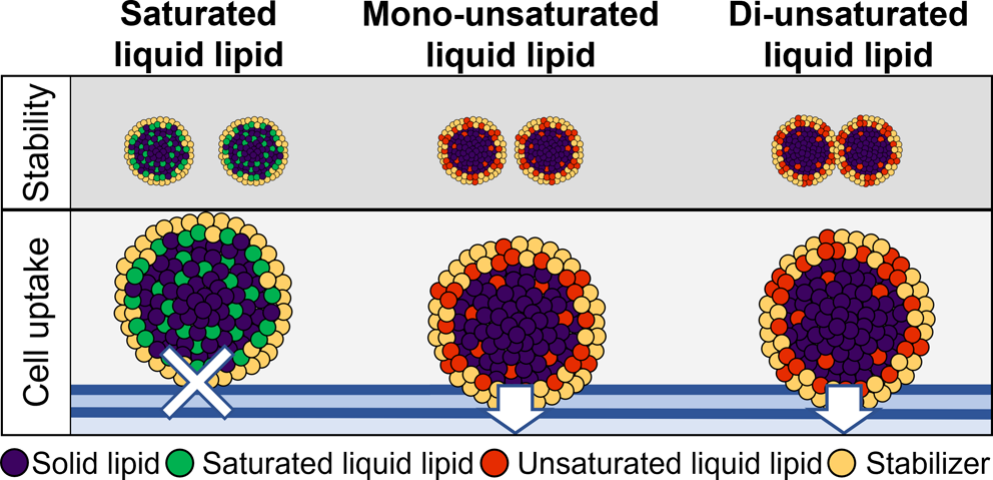

The efficacy of nanostructured lipid carriers (NLC) for drug delivery strongly depends on their stability and cell uptake. Both properties are governed by their compositions and internal structure. To test the effect of the lipid composition of NLC on cell uptake and stability, three kinds of liquid lipids with different degrees of unsaturation are employed. After ensuring homogeneous size distributions, the thermodynamic characteristics, stability, and mixing properties of NLC are characterized. Then the rates and predominant pathways of cell uptake are determined. Although the same surfactant is used in all cases, different uptake rates are observed. This finding contradicts the view that the surface properties of NLC are dominated by the surfactant. Instead, the uptake rates are explained by the structure of the nanocarrier. Depending on the mixing properties, some liquid lipids remain inside the nanocarrier, while other liquid lipids are present on the surface. Nanocarriers with liquid lipids on the surface are taken up more readily by the cells. This shows that the engineering of efficient lipid nanocarriers requires a delicate balance of interactions between all components of the nanocarrier on the molecular level.

## Introduction

Significant advances in materials science have led to the development of improved nanosystems for targeted and controlled drug delivery. With several approved products on the market, the research of nanosystems in medicine is shifting to optimizing efficacy and safety.^1,2^ To achieve this, a fundamental understanding of the interactions of nanomaterials with target cells is required.^3^ The physicochemical properties and structure of nanosystems are decisive for these interactions.^1,4−6^ Numerous reviews have aimed to establish a universally applicable strategy for predicting cellular interactions based on nanoparticle attributes.^7−10^ While it has been postulated that the size of nanoparticles determines the endocytic uptake pathway, different trends were observed depending on the nanomaterials and target cells considered.^11,12^ Surface composition and charge are further critical characteristics, as they dictate interactions with body fluids, the protein corona formation and composition, the *in vivo* biodistribution, the excretion dynamics from the body, and the efficacy and toxicity profile of cellular uptake.^13−19^ It is worth noting that positively charged particles have an increased absorption capacity, albeit at the expense of increased toxicity.^7,20^ Nevertheless, the most important factor for the interaction between nanomaterials and cells is the chemical nature of the materials.^7^

Modern nanosystems exhibit increasing structural complexity, which poses a challenge to predictions of *in vivo* performance. Experimental protocols to determine the properties and structure of nanoparticles are well established, but most experiments are limited in terms of spatial and temporal resolution.^14^ In such cases, molecular dynamics simulations can capture the structures of nanosystems on a molecular level. Here, the well-established MARTINI force field is used, which groups three to four heavy atoms into beads, and explicitly considers water and ions.^21^ This enables the characterization of large and complex nanosystems on a microsecond time scale.

To study the impact of the nanoscale structure on the cell uptake and stability, nanostructured lipid carriers (NLC) consisting of the solid lipid (i.e., high melting lipid—solid at room and body temperature) Compritol 888 ATO (C), the stabilizer Tween 80 (Tween 80), and liquid lipids (i.e., low melting lipids—liquid at room and body temperature) are used. Three different liquid lipids with different degrees of saturation are selected: middle-chain triglycerides (MCT, saturated), oleic acid (OA, one double bond), and linoleic acid (LA, two double bonds). The mixtures are tested for processability, stability, biocompatibility, and cellular uptake. The oral mucosa was selected as target tissue to investigate the applicability of NLC for the local treatment of oral mucositis and xerostomia, thus enabling the optimization of the systems. For this purpose, buccal epithelial TR146 cells were selected as a model cell line. First, Design of Experiment (DoE) studies are conducted to enable the production of NLC with uniform size distributions. Based on Teubl et al.,^22,23^ particle sizes between 200 and 300 nm with a negative surface charge are used. This allows a systematic investigation of the cellular uptake based on the contribution of the liquid lipid.^24^ The homogeneity of the particles with regard to size distribution, surface charge, and internal miscibility is validated experimentally before testing the cellular uptake with epithelial cells of the oral mucosa. Coarse-grained molecular dynamics simulations are used to determine the distribution of the individual components inside the NLC, which allows an interpretation of the experimental data.

## Experimental Section

### Materials

Compritol 888 ATO (C; USP NF Name: Glyceryl dibehenate) was provided by Gattefossé (Saint Priest, France). It consists of mono-, di-, and triesters of behenic acid (C22) with the diester fraction being the predominant. Oleic acid (OA) was provided by Croda GmbH (Nettetal, Germany). Medium-chain triglycerides (MCT) were purchased from Herba Chemosan Apotheker-AG (Vienna, Austria). Linoleic acid (LA), Tween 80 (Tween 80), oil-red-o, hydrogen peroxide (H_2_O_2_), dynasore hydrate, chlorpromazine hydrochloride, genistein, 5-(*N*-ethyl-*N*-isopropyl)amiloride (EIPA), and MEM nonessential amino acid solution (100×; NEAA) were obtained from Sigma-Aldrich (Munich, Germany). Human buccal TR146 cells from Imperial Cancer Research Technology (London, U.K.) were used for all cell culture experiments. Dulbecco’s Modified Eagle’s medium (DMEM), phosphate buffered saline (PBS; pH 7.4), fetal bovine serum (FBS), penicillin streptomycin (Penstrep), Hank’s Balanced Salt Solution (HBSS), and 0.25% trypsin-ethylenediaminetetraacetic acid (trypsin-EDTA) were obtained from Gibco, Life Technologies Corporation (Painsley, U.K.). Dihydroethidium (DHE), Alexa Fluor 488 Phalloidin, and Hoechst 33342 were purchased from Thermo Fisher Scientific (Vienna, Austria). HyClone (i.e., serum-free DMEM) was obtained from GE Healthcare Life Sciences (Logan). Ultrapurified water (i.e., Milli-Q-water (MQ-water); Millipore SAS, Molsheim, France) was used for all experiments.

### Preparation of NLC with Defined Characteristics Using DoE

The different formulations (i.e., C/MCT, C/OA, and C/LA) of NLC were prepared via the hot high-pressure homogenization (HPH) approach preceded by a hot emulsification step using high-shear mixing (HSM), followed by cooling. DoE studies using the Modde 13.0 program (Satorius AG, Göttingen, Germany) were conducted to prepare nanoparticles with defined sizes between 200 and 300 nm with a narrow particle size distribution (i.e., Polydispersity Index (PdI) <0.3). The effects of the number of HPH cycles, HPH pressure, stabilizer concentration, and lipid matrix composition were investigated. A central composite face design including three center points and a quadratic model type was applied. In total, 27 experiments were conducted for each type of liquid lipid. The ranges of the parameters were selected based on previous experiments (three to ten HPH cycles, HPH pressures of 250–750 bar, stabilizer concentrations of 1–4% (w/w), and solid lipid to liquid lipid ratios of 9:1, 8:2, and 7:3 (w:w)). Other parameters like batch size (75 g) and total lipid concentration (10% w/w) were fixed. The results from particle size analyses via laser diffraction (LD) (i.e., d (0.9) values) were set as response and were fitted as a function of the factors using multiple linear regression.

For the preparation of the different NLC formulations, the lipid phase consisting of the solid lipid and the respective liquid lipid was heated 10 °C above the *T*_m_ of the solid lipid (i.e., 75 °C; process temperature = 85 °C). The aqueous phase comprising MQ-water and Tween 80 was heated to the same temperature before the addition to the lipid phase. Subsequently, HSM (Ultra Turrax T25 digital equipped with S25N-18G, IKA-Labortechnik, IKA-Werke GmbH & Co., KG Staufen, Germany) of the premix was performed at 12,000 rpm for 30 s. The resulting pre-emulsion was transferred into a piston gap high-pressure homogenizer (Panda 2K, NS1001L Spezial, GEA Niro Soavi, Lübeck, Germany) with an external water bath for temperature control. The emulsions were collected in glass vials and cooled. Preliminary studies showed that C/MCT formulations are stable at room temperature, while C/OA and C/LA formulations need to be cooled to 5 ± 3 °C. For cellular uptake studies, fluorescence labeled NLC were prepared by replacing 2% (w/w) of the lipid phase with the dye oil-red-o.

### Characterization of NLC

#### Particle Size and ζ-Potential

The particle sizes were investigated via LD using the Mastersizer 2000 with the Hydro 2000 μp-unit (Malvern Instruments, Malvern, U.K.). MQ-water with a refractive index (RI) of 1.330 was used as the measurement medium. The real RI and the imaginary RI of the samples were set to 1.360 (solid lipid) and 0.001. NLC were added until an obscuration range between 4 and 6% was reached. A stirrer speed of 1750 U/min was applied, and measurements were performed in triplicates. Analysis of the samples was conducted via Mie theory. The results were presented as volume-based *d* (0.1), *d* (0.5), *d* (0.9), d (0.95), and *d* (0.99) values, which indicate that 10, 50, 90, 95, and 99% of the particles are smaller than the specified size.

The particle size of NLC was further examined via dynamic light scattering (DLS) using a Zetasizer Nanoseries Nano ZS (Malvern Instruments) with a 633 nm laser. NLC were diluted with MQ-water to avoid multiple scattering. The RIs were set to 1.330 for MQ-water as a dispersant and 1.360 (imaginary RI of 0.001) for NLC. Each sample was measured in triplicate at 25 °C using a measurement angle of 173° (backscatter) and an equilibration time of 30 s. The measurement duration was set automatically by the device, and mean hydrodynamic diameters (*Z*-average) and the PdI, which is a measure of the width of the distribution, were determined.

The ζ-potential was investigated via electrophoretic light scattering (ELS, Zetasizer Nano ZS, Malvern Instruments). Samples were diluted with zeta-water (MQ-water adjusted with 0.9% (w/v) sodium chloride solution to a pH of 5.5–6 and a conductivity of 50 μS/cm^25^). Measurements were conducted in triplicate at 25 °C after an equilibration time of 30 s, and scattered light was detected at an angle of 173°. The measurement duration was set automatically by the device (minimum of 10 and maximum of 100 runs). Calculation was performed according to the Helmholtz-Smoluchowski equation. To determine the physical stability, the particle size and ζ-potential of the samples were monitored on a weekly basis over a period of 4 weeks via LD, DLS, and ELS. In addition, the particle size and PdI of NLC diluted in serum-free DMEM at various concentrations (100–1000 μg/mL) was monitored over a period of 4 h at 37 °C.

#### Miscibility Studies Using Differential Scanning Calorimetry (DSC)

The thermodynamics of the bulk materials, the binary mixtures comprising the liquid lipids and the stabilizer and the solid lipid and the liquid lipids, the ternary mixtures, and the air-dried NLC were investigated via DSC (204F1 Phoenix, Netzsch GmbH, Selb, Germany). Three to ten mg of the respective samples were placed into aluminum crucibles and sealed with pierced lids. The DSC cell was purged with pure nitrogen at a flow rate of 20 mL/min. As a reference material, an empty aluminum crucible was used. The samples were heated from −20 to 100 °C at a heating/cooling rate of 10 °C/min. Samples were investigated in triplicate, and data were analyzed using the NETZSCH Proteus software (Netzsch GmbH).

### Coarse-Grained Molecular Dynamics Simulations to Simulate the NLC Structure

The Martini Force Field 2.0^26,27^ with polarizable water was used to model all system components. Structures and standard parameters for the C, MCT, OA, and LA were adapted from Marrink et al.^26^ and Tween 80 from Luz et al.^28^ A slab of NLC was simulated, thus employing the spherical symmetry of NLC to reduce the computational costs. The initial volume of the slab was 42.4 × 42.4 × 260 nm^3^. The short box side lengths were set to 42.4 nm to reduce the potential error between the arc (full NLC sphere) and line (rectangular slab) below 1%. Phase profiles from the center to the surface were obtained using the species number density. The box contained a central layer of solid lipids bounded on either side by a layer of liquid lipids, followed by the stabilizer layer and a 20 nm layer of polarizable water. The number of molecules in each layer matched the experimental molar ratios of the NLC. The exact species numbers are listed in Table 1 of the Supporting Information. The systems were generated using the software package Packmol.^29^

All simulations were conducted using the GROMACS 2023.1 package.^30,31^ All runs were performed at 277 K, the storage temperature for the NLC (i.e., 4 °C), using a velocity rescale thermostat.^32^ The energies were minimized with steepest descent for 10,000 steps and equilibrated for 200 ns with a 10 fs time step and a Parrinello–Rahman^33,34^ semi-isotropic barostat. The compressibility was set to 3 × 10^–4^ bar^–1^ and the pressure was set to 1 bar. Production runs were 2 μs. Lennard-Jones interactions used a potential-shift-Verlet scheme with radii of 0.9 and 1.2 nm. The reaction field method was used to treat electrostatic interactions beyond the 1.2 nm cutoff. Radial distribution functions were calculated using the gmx density tool of GROMACS. The results were averaged over four production simulations using periodic boundary conditions.

### Cell Culture

Cultivation of TR146 cells was conducted in DMEM supplemented with 10% FBS, 1% NEAA, and 1% PenStrep at 37 °C in a humidified atmosphere containing 5% CO_2_. The medium of the cells was changed two to three times per week. Subcultivation of confluent cells was performed once a week, using 0.25% trypsin-EDTA. Serum-free DMEM without phenol red supplemented with 1% NEAA and 1% PenStrep was used for all experiments.

#### Cell Uptake Studies

TR146 cells were seeded in 8-well chamber glass slides (Szabo-Scandic, Corning, Vienna, Austria) using a seeding density of 1.5 × 10^4^ cells/well and were cultured for 1 week. For visualization, cells were incubated with oil-red-o labeled NLC diluted with serum-free DMEM to reach final concentrations of 500 and 750 μg/mL in a time-dependent manner (i.e., 30 min, 1, 2, and 4 h). Untreated cells were used as a control and were incubated with serum-free DMEM. After the incubation period, cells were washed with PBS and the cytoskeleton was stained with Alexa Fluor 488 Phalloidin. The cell nuclei were counterstained with Hoechst 33342. Acquisition of images was performed with a confocal laser scanning microscope LSM 510 Meta (cLSM; Carl Zeiss GmbH, Vienna, Austria) equipped with the ZEN2008 software package. Oil-red-o labeled NLC were visualized at an excitation wavelength of 633 nm using a LP 650 nm long pass detection for the red channel. The cytoskeleton was detected at an excitation wavelength of 488 nm using a bandpass (BP) of 505–550 nm for the green channel region and the cell nuclei at 405 nm with a BP of 420–480 nm bandpass detection for the blue channel. Images of randomly chosen areas of the cell monolayers were recorded via a cLSM. *Z*-stacks were acquired, and virtual radial sections were documented. To identify the involved mechanism, the cells were incubated with PBS and 200 μM dynasore, 20 μM chlorpromazine, 300 μM genistein or 0.5 μM EIPA diluted in serum-free DMEM, and handled as described earlier.^35^

For spectral fluorescence cytometry experiments, TR146 cells were seeded in 24-well polystyrene plates (Greiner Bio-One GmbH, Frickenhausen, Germany) using a seeding density of 4 × 10^4^ cells/well and were cultured for 1 week. Cells were washed with PBS and incubated with oil-red-o labeled NLC diluted with serum-free DMEM to reach final concentrations of 500 and 750 μg/mL for 4 h. The medium was removed, and cells were washed with PBS and detached with 100 μL of 0.25% trypsin-EDTA per well. After an incubation time of 5 min, the reaction was stopped with 900 μL of serum-free DMEM. The cells were centrifuged for 5 min at 400 x *g* (Centrifuge 5417 R, Eppendorf Austria GmbH, Vienna, Austria). The supernatant was discarded, and the cells were resuspended in 200 μL of PBS and further used for flow cytometry analysis (Cytek aurora, Cytek Biosciences).

For colocalization studies, TR146 cells with a seeding density of 6 × 10^4^ cells were cultured in 24-well glass bottom plates (Porvair Sciences, Wrexham, U.K.) for 1 week. Cells were washed with PBS and incubated with NLC diluted in a serum-free medium at concentrations of 500 and 750 μg/mL for 2 and 4 h, respectively. Subsequently, endoplasmic reticulum (ER), mitochondria, and lysosomes were stained using 1 μM ER-Tracker Green (Thermo Fisher Scientific, Vienna, Austria), 100 nM Mito Red (Sigma-Aldrich, Munich, Germany), and 50 nM LysoTracker Red DND-99 (Thermo Fisher Scientific, Vienna, Austria) according to the instructions. After an incubation time of 30 min, cell nuclei were counterstained with Hoechst 33342 for 10 min. Particle uptake was visualized using a Nikon Eclipse Ti2 microscope (Nikon GmbH, Vienna, Austria) equipped with an Andor Zyla sCMOS camera. Cell nuclei were detected at 395 nm excitation and 432 nm emission wavelength. Mitochondria and lysosomes were visualized at 555 nm excitation wavelength and 595.5 nm emission wavelength, and the ER was visualized at 470 nm excitation wavelength and 515 nm emission wavelength. Oil-red-o labeled nanoparticles were detected at 640 nm excitation wavelength and 730.5 nm emission wavelength. The uptake was evaluated using NIS-Elements 5.21.03 software.

### Statistical Analysis

If not otherwise stated, experiments were performed in triplicate, and results were presented as mean values ± standard deviation (SD). Statistical analyses were conducted via Student’s *t* tests. Differences were considered to be significant at a level of *p* ≤ 0.05 (*), *p* ≤ 0.01 (**), and *p* ≤ 0.001 (***).

## Results and Discussion

### Preparation of NLC with Defined Characteristics Using DoE

As a first step, to avoid artifacts in the cell uptake studies, production conditions are screened to yield uniform particle sizes between 200 and 300 nm for all three kinds of NLC.^24^ Initial parameter-screens yield size distributions with d (0.9) sizes between 215 and 240 nm for C/MCT-NLC, and 300 to 700 nm for C/OA- and C/LA-NLC. No outliers (i.e., ±4 SD) are detected in the normal probability plots, and all experiments exhibit a linear distribution, which allows the creation of a linear model to guide the search for suitable parameters (see Supporting Information, Supporting Figure 1). The established models exhibit *R*^2^ values of 0.810 (C/MCT), 0.924 (C/OA), and 0.945 (C/LA), indicating a very good fit of the regression model (see Supporting Information, Supporting Figure 2). Further center point experiments indicate high reproducibility (i.e., 99.9%) and robustness of the model for all formulations.

The coefficients of the model (see Supporting Figure 2) reveal that the stabilizer concentration and its quadratic effects are the most significant factors affecting the particle size, independent of the matrix composition. This is in accordance with previous findings in the literature.^36,37^ Higher stabilizer concentrations are associated with smaller particle sizes, but, due to the opposite signs of the linear and quadratic terms, only until a saturation point.^38,39^ For C/MCT formulations, besides the number of cycles, a 9:1 (w:w) ratio of solid-to-liquid lipid leads to significantly smaller particles. Furthermore, a strong interdependence between the pressure and stabilizer is observed. This correlation is also evident in the C/OA and C/LA formulations. Although a high number of cycles and a high pressure generally lead to a reduction in particle size, overprocessing poses the risk of increasing particle size for C/OA- and C/LA-NLC. Also, the interaction between stabilizer and the lipid ratio affects the size of C/OA- and C/LA-NLC, which is influenced by factors such as HLB values, material miscibilities, and molecular geometry.^38,40,41^ Based on the results, eight cycles at 500 bar and a stabilizer concentration of 2.5% (w/w) are chosen for all three formulations. The use of a moderate stabilizer concentration also mitigates the risk of cytotoxic effects associated with the stabilizer.^40,42^

### Characterization of NLC

#### Particle Size and ζ-Potential

The optimized formulations from the previous section show only small size variations in DLS experiments with respect to the degree of unsaturation in the liquid lipid (MCT < OA < LA). The *Z*-average size is 217 ± 1 nm for C/MCT-NLC, 224 ± 6 nm for C/OA-NLC, and 261 ± 6 nm for C/LA-NLC. The PdI values are consistently low, ranging between 0.222 ± 0.016 for C/MCT-, 0.219 ± 0.004 for C/OA-, and 0.215 ± 0.005 for C/LA-NLC. LD measurements agree with the DLS findings, showing *d* (0.9) values of 212 ± 0 nm for C/MCT-, 219 ± 1 nm for C/OA-, and 227 ± 2 nm for C/LA-NLC at day zero. The *d* (0.95) values remain below 280 ± 0 nm for all formulations, which shows that less than 5% of the particles form agglomerates. Likewise, the addition of the dye oil-red-o for the uptake experiments has a negligible impact on the particle size, resulting in *Z*-average sizes of 189 ± 1 nm for C/MCT-, 228 ± 4 nm for C/OA-, and 251 ± 2 nm for C/LA-NLC. Similarly, the PdI values remain consistent, measuring 0.198 ± 0.002 for C/MCT-, 0.197 ± 0.008 for C/OA-, and 0.212 ± 0.010 for C/LA-NLC.

To assess the stability of the colloidal dispersion in terms of electrostatic repulsion between the particles, the ζ-potentials are determined. The ζ-potential measurements in zeta-water range from −21.6 ± 0.6 to −35.6 ± 0.7 mV, confirming physically stable formulations.^43^ After dilution of the dispersions with serum-free medium and subsequent incubation at 37 °C for 4 h, no significant changes in colloidal stability are observed. Stability studies performed over 28 days of storage at room temperature (C/MCT-NLC) and 5 ± 3 °C (C/OA- and C/LA-NLC) show that C/MCT formulations remain stable with a *Z*-average size of 222 ± 5 nm, while C/OA-NLC increases slightly to 248 ± 5 nm, which is consistent with LD data (i.e., *d* (0.9) values of 216 ± 0 nm for C/MCT- and 225 ± 0 nm for C/OA-NLC, see Supporting Information, Supporting Figure 3). No significant changes in size and constant ζ-potential (−21.7 ± 2.3 mV for C/MCT- and −30.53 ± 0.21 mV for C/OA-NLC) are observed. In contrast, C/LA formulations gel after 2 weeks, revealing a notable change in physical properties.

#### Miscibility Studies Using Differential Scanning Calorimetry (DSC)

**Figure 1:**
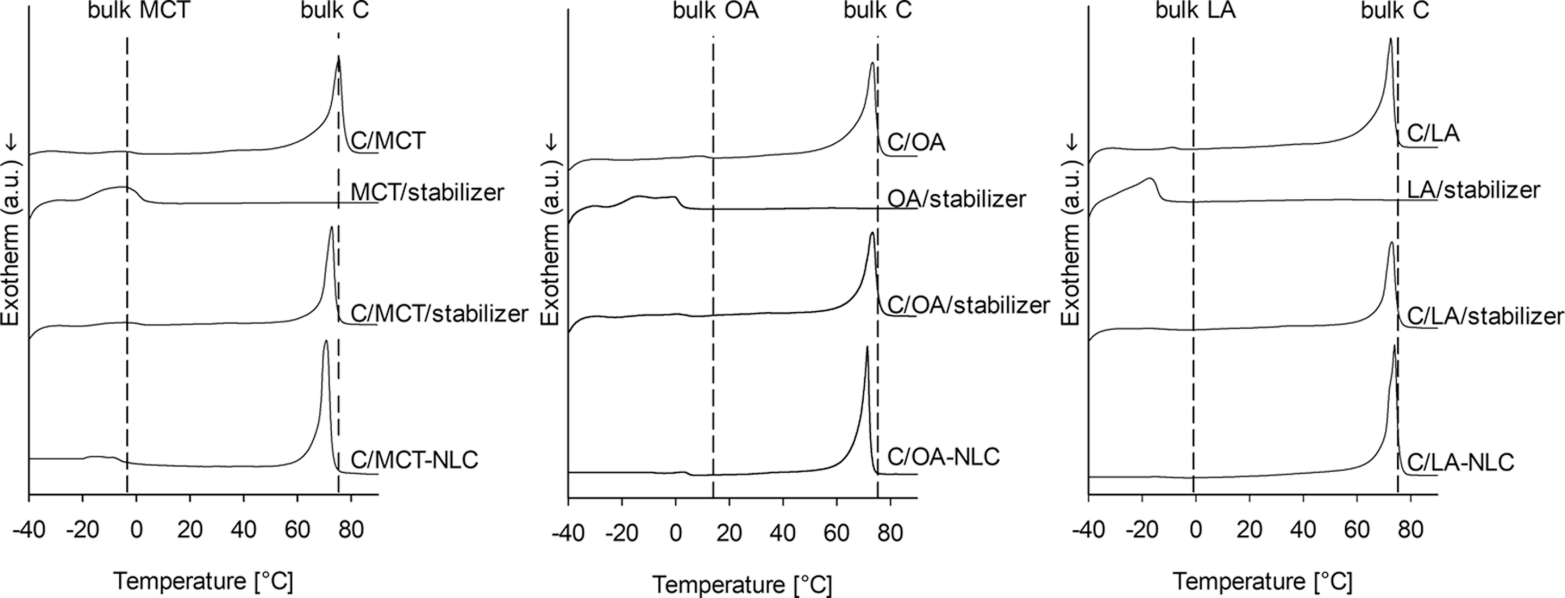
DSC thermograms of lipid mixtures and NLC formulations. The thermograms of the binary physical mixtures of the solid lipid and the liquid lipids (i.e., C/MCT; C/OA; C/LA) are shown on the top, followed by the thermograms of the binary physical mixtures of the liquid lipids and the stabilizer (i.e., MCT/stabilizer, OA/stabilizer, LA/stabilizer) and of the ternary physical mixtures of the solid lipid, the liquid lipids, and the stabilizer (i.e., C/MCT/stabilizer, C/OA/stabilizer, C/LA/stabilizer). At the bottom, the thermograms of the air-dried C/MCT-NLC, C/OA-NLC, and C/LA-NLC formulations are presented. The vertical dashed lines correspond to the melting points of the respective bulk liquid lipids (i.e., bulk MCT, OA, and LA) and the bulk solid lipid (bulk C).

To investigate the interactions between the individual components of the mixtures, DSC experiments are conducted. The thermal events, including melting and recrystallization temperatures, of the pure substances align with literature values^44−47^ (see Supporting Information, Supporting Figure 4). In the binary mixtures of solid and liquid lipids, the thermal events associated with the liquid lipids are consistently observed across all three mixtures, indicating incomplete mixing (see Figure 1, C/MCT, C/OA, C/LA).^48,49^ A shift in the onset or melting peak of the solid lipid (C) relative to the bulk material is observed upon mixing with the unsaturated liquid lipids (i.e., melting temperature (*T*_m_) = 72.8 ± 0.6 °C with an onset at 69.8 ± 0.2 °C for C/OA, *T*_m_ = 72.5 ± 0.2 °C with an onset at 69.7 ± 0.1 °C for C/LA). Likewise, the peaks of all three liquid lipids (i.e., C/MCT *p* = 0.014, C/OA *p* < 0.001, C/LA *p* < 0.001) shift toward lower temperatures, which suggests at least partial miscibility. Mixing the stabilizer with the individual liquid lipids leads to a shift (∼15 °C) of the melting peaks to lower temperatures exclusively for the unsaturated lipids (i.e., OA/stabilizer *p* < 0.001 and LA/stabilizer *p* < 0.001) (see Figure 1, MCT/stabilizer, OA/stabilizer, LA/stabilizer). This indicates increased interactions between the unsaturated lipids and the stabilizer compared to the saturated one, which is also confirmed by the results of the miscibility studies based on the Gibbs transfer free energy (see Supporting Information, Supporting Figure 6).

In the three-component physical mixtures, the melting peak of C in the C/MCT/stabilizer mixture (*T*_m_ = 73.8 ± 1.1 °C with an onset at 68.8 ± 0.1 °C) is only slightly shifted (*p* = 0.098) to lower temperatures compared with the bulk material, and the MCT peak becomes more discernible and broader relative to the bulk MCT, which again indicates partial miscibility of the components. For the C/OA/stabilizer and C/LA/stabilizer, the thermal events associated with all lipids shift to lower temperatures (shift of *T*_m_ of C with *p* = 0.010 for the C/OA/stabilizer and *p* = 0.002 for the C/LA/stabilizer, respectively). The higher miscibility of the stabilizer with the unsaturated lipids is further supported by Raman measurements (see Supporting Figure 5). In air-dried NLC formulations, the thermal events of the liquid lipids are evident, accompanied by a significant decrease in the *T*_m_ of the solid lipid for all three matrix compositions (*T*_m_ = 73.3 ± 0.6 °C with an onset at 70.2 ± 0.3 °C for C/MCT-NLC, *p* = 0.011; *T*_m_ = 71.3 ± 0.1 °C with an onset at 68.7 ± 0.1 °C for C/OA-NLC, *p* < 0.001; and *T*_m_ = 73.3 ± 0.9 °C with an onset at 71.0 ± 0.2 °C for C/LA-NLC, *p* = 0.018). This indicates a decrease in the packing density of the solid lipid, which provides additional space for the incorporation of active pharmaceutical ingredients.^48−51^

### Coarse-Grained Molecular Dynamics Simulations to Simulate NLC Structure

**Figure 2:**
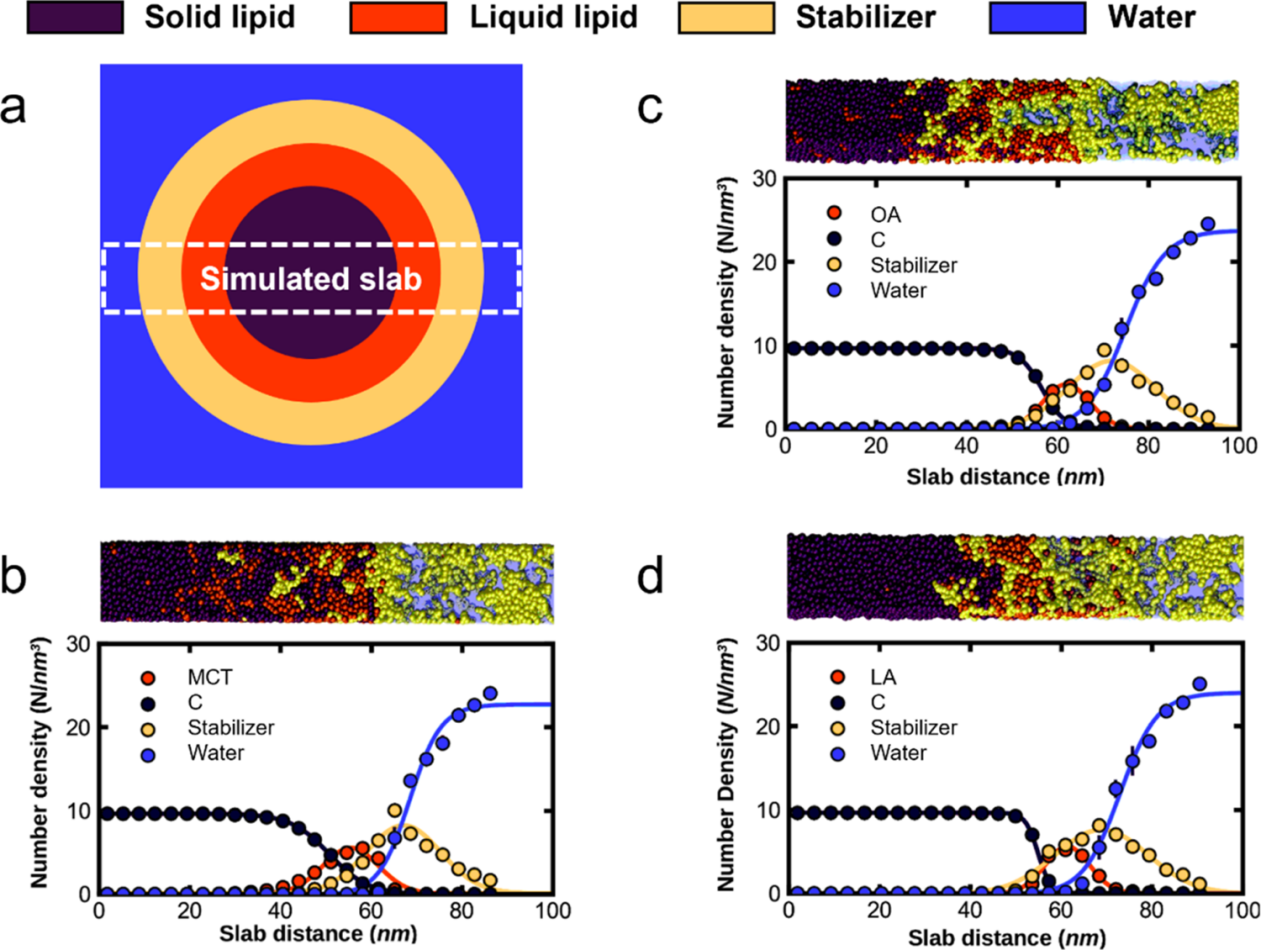
Sketch of the slab geometry used in the simulations with respect to the whole NLC (a) and snapshots (top) and density profiles (bottom) along a cross-section through NLC consisting of three different liquid lipid species (i.e., (b) MCT, (c) OA, and (d) LA). The phase profiles are fitted to error functions (continuous lines). The error bars are the standard error from the averaged set of eight samples per liquid lipid species.

To determine the internal structure of the NLC, density profiles of the different components are calculated from the coarse-grained molecular dynamics simulations (see Figure 2). The density profiles show defined phases for each NLC component, starting with the solid lipid on the inside of the NLC (dark purple, C), followed by the liquid lipid (orange), stabilizer (yellow), and, outside of the NLC, water (blue). The solid lipid maintains a well-defined density plateau with the concentration dropping to zero at around 60 nm. As the degree of unsaturation of the liquid lipid increases (MCT < OA < LA), the plateau of the solid lipid extends further, indicating slightly larger particle sizes, as observed in the LD and DLS experiments. In addition, the interface between the solid and the liquid lipid becomes sharper (MCT < OA < LA). This indicates decreasing miscibility between the solid and liquid lipid with increasing degree of unsaturation, which agrees with the DSC and Raman data.

In contrast, the phase boundary between the liquid lipid and the stabilizer becomes less sharp with an increasing degree of unsaturation. The stabilizer peak broadens, and the interfacial region with the liquid lipid shows a higher stabilizer density. It is also found that the Gibbs transfer free energy of the liquid lipid from its bulk phase to the stabilizer phase decreases with increasing degree of unsaturation (see Supporting Information, Supporting Figure 6). Both results indicate increasing miscibility of the liquid lipid with the surfactant with an increasing degree of unsaturation, which agrees with the DSC and Raman data. The interactions of the main components of the NLC surface are further characterized by radial distribution functions from the simulations (see Supporting Information, Supporting Figure 7), showing that the hydrophobic tail of the surfactant favors interactions with the liquid lipid, while the hydrophilic tails of the surfactant mostly remain in contact with water. Notably, all simulation data show that the exposure of the liquid lipid to water on the surface increases with increasing degree of unsaturation.

### Cell Uptake Studies

**Figure 3:**
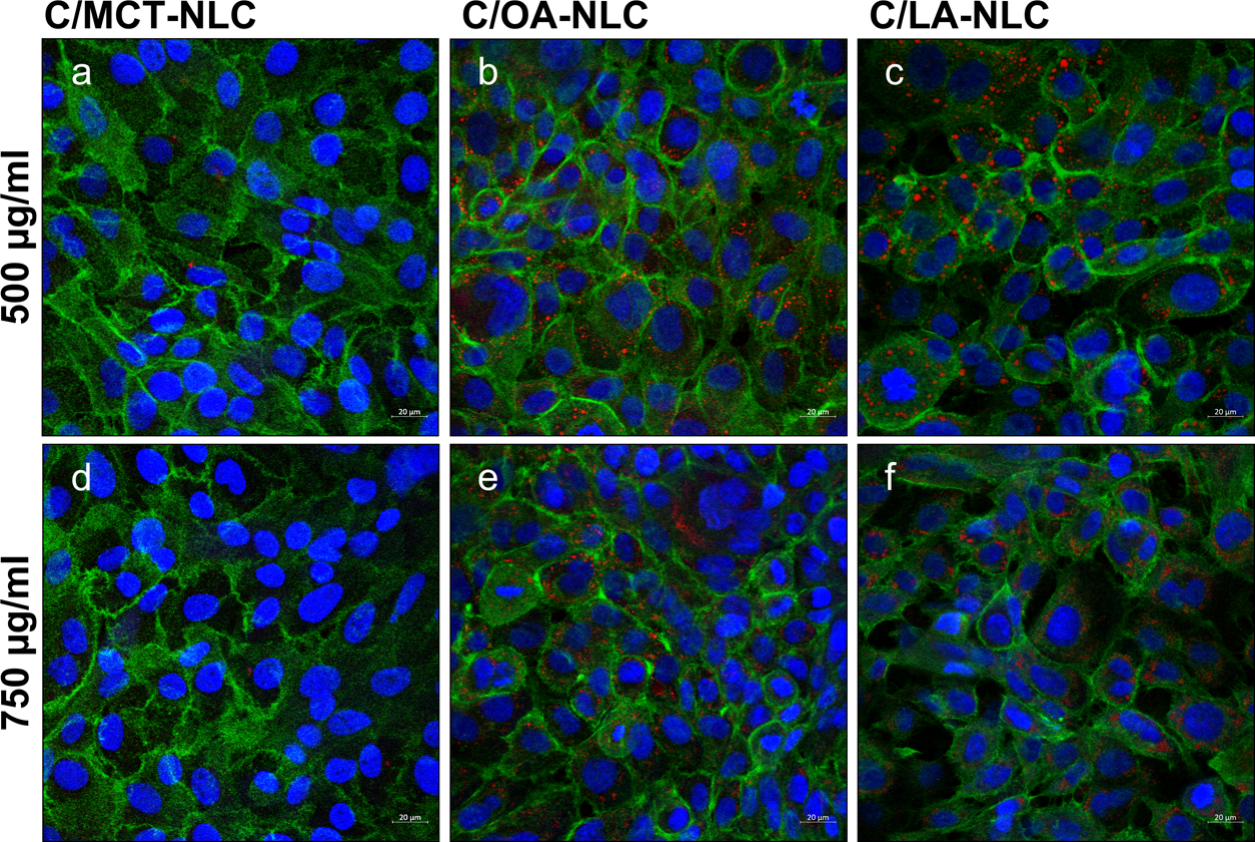
Fluorescence microscopic images of cellular uptake studies of oil-red-o labeled NLC (i.e., C/MCT- (a, d), C/OA- (b, e), and C/LA-NLC (c, f); red) in TR146 cells using concentrations of 500 (a–c) and 750 μg/mL (d–f). Cell nuclei were stained with Hoechst 33342 (blue), and cytoskeleton was stained with Alexa Fluor 488 Phalloidin (green). Red dots inside of the cells indicate NLC uptake. Scale bar = 20 μm.

Figure 3 shows the uptake behaviors of all three NLC types. C/MCT-NLC are not taken up by the cells within 4 h, regardless of the concentration. The spectral flow cytometry experiments after 4 h of incubation at a concentration of 500 μg/mL show that the fluorescence signal increases as a function of the degree of unsaturation (i.e., 3.2 ± 0.1-fold for C/MCT-NLC, 9.6 ± 0.7-fold for C/OA-NLC, and 10.2 ± 1.2-fold for C/LA-NLC, relative to control). With increasing concentration (750 μg/mL), the uptake increases further (i.e., 3.9 ± 0.3-fold for C/MCT-NLC, 16.0 ± 1.6-fold for C/OA-NLC, and 20.4 ± 1.6-fold for C/LA-NLC, relative to the control). This result is explained by the simulations, where MCT is not present at the NLC surface, which hinders a fusion with the cell membrane.^18^ Conversely, the increased miscibility of OA and LA with the surfactant leads to higher lipid concentrations on the surface, which facilitates fusion events with the cell membrane. During fusion, the unsaturated lipids make the membrane more fluid, which increases the particle flux.^18,52,53^ The exact position of the double bond of the unsaturated lipid is crucial for the structure of the membrane, as it affects the bending and conformational entropy of the hydrocarbon chain.^54^ Both OA and LA contain one double bond at position 9,10, while a second double bond is located at 12,13 in LA. Double bonds at positions 9,10 or 10,11 are reported to have stronger effects than those at other positions.^55,56^ This explains why the uptake of LA is only slightly increased compared to that of OA.

**Figure 4:**
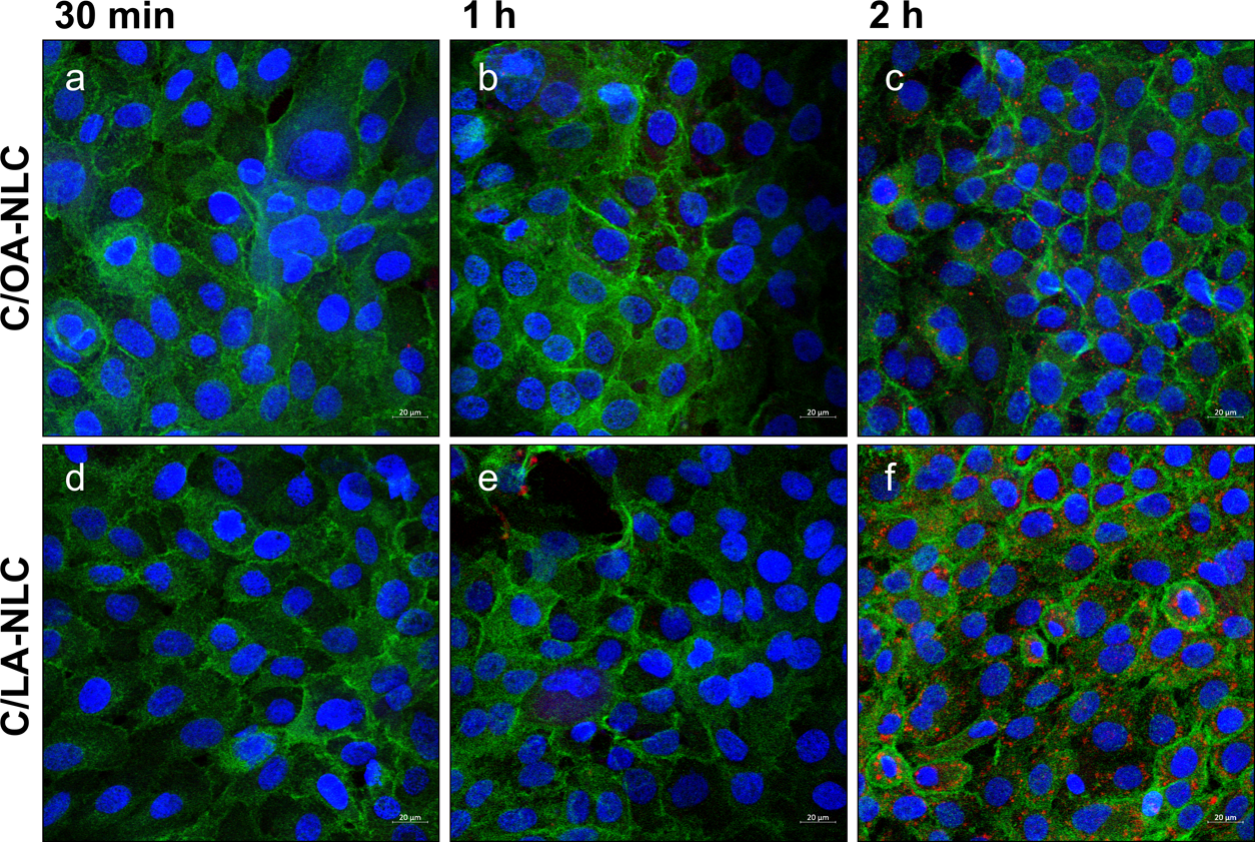
Fluorescence microscopic images of cellular uptake of oil-red-o labeled NLC (i.e., C/OA- (a–c) and C/LA-NLC (d–f); red) in TR146 cells at a concentration of 750 μg/mL after 30 min (a, d), 1 h (b, e), and 2 h (c, f) of incubation. Cell nuclei were stained with Hoechst 33342 (blue), and cytoskeleton was stained with Alexa Fluor 488 Phalloidin (green). Red dots inside of the cells indicate NLC uptake. Scale bar = 20 μm.

Figure 4 shows the time-dependent uptake of C/OA- and C/LA-NLC. After 30 min of incubation, no particles are detected in the cells. After 1 h (Figure 4b,e), uptake is minimal in both cases, and after 2 h (Figure 4c,f), a significant increase in NLC concentration is observed in the cells. Cellular uptake of nanocarriers via endocytosis only occurs if nanoparticles are able to sediment on the cell′s surface, which in turn depends on bulk fluid flow.^57^ Therefore, it is likely that in addition to the size and matrix composition, the biomechanical properties (e.g., density, stiffness) of NLC together with the environmental conditions (i.e., saliva in healthy versus diseased state) are critical for particle floating or sedimentation and thus determine cellular uptake.

**Figure 5:**
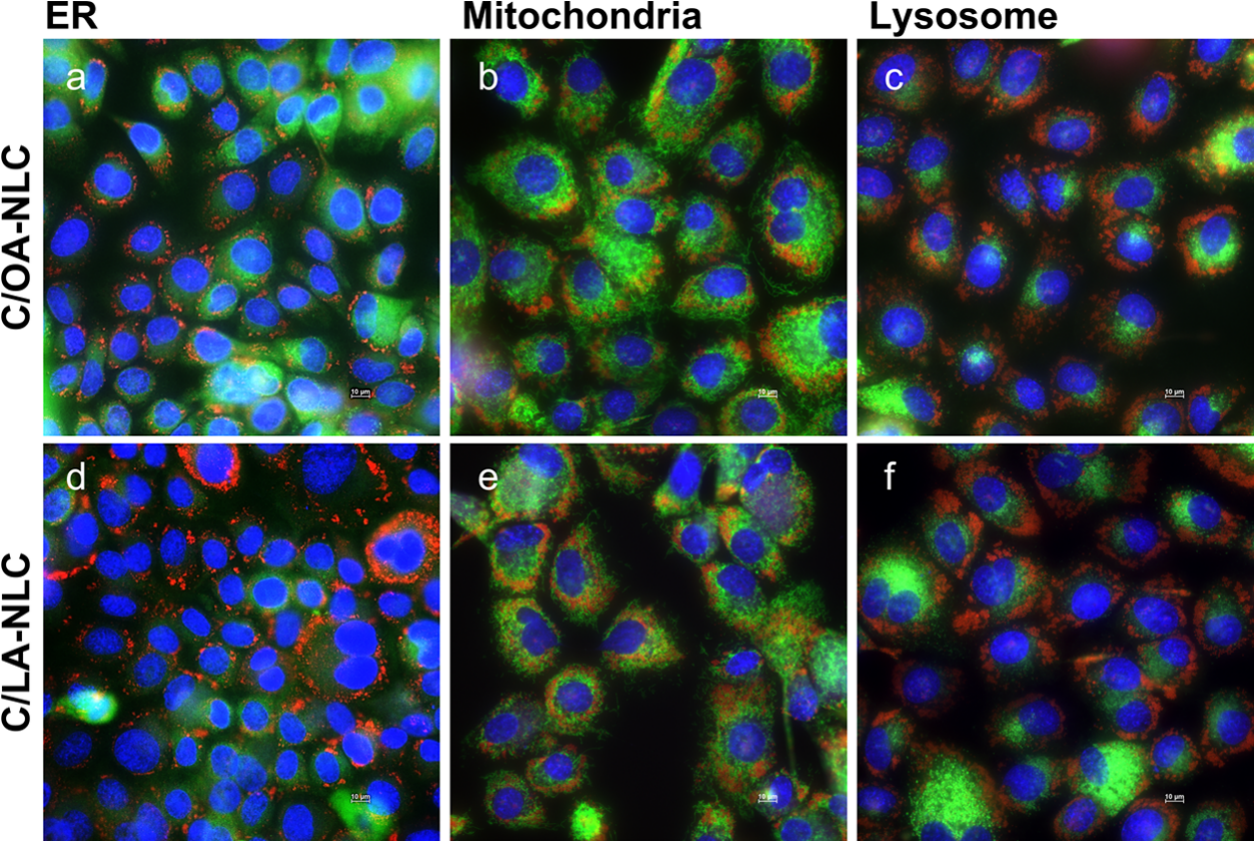
Fluorescence microscopic images of cellular uptake of oil-red-o labeled NLC (i.e., C/OA- (a–c) and C/LA-NLC (d–f); red) in TR146 cells at a concentration of 750 μg/mL after an incubation time of 4 h. Endoplasmic reticulum (ER, a, d), mitochondria (b, e), and lysosome (c, f) are visualized in green. Cell nuclei were stained with Hoechst 33342 (blue). Most NLC (red dots) are located near the ER or the mitochondria. Scale bar = 10 μm.

Blocking the clathrin-mediated endocytosis pathway results in no significant changes of the uptake signal for both formulations (i.e., 13.4 ± 0.1-fold increase for C/OA-NLC and 18.9 ± 0.9-fold increase for C/LA-NLC), compared to the noninhibited state (i.e., 16.0 ± 1.6-fold increase for C/OA-NLC and 20.4 ± 1.6-fold increase for C/LA-NLC). Inhibition of macropinocytosis leads to a significant reduction in cellular uptake of C/LA-NLC (i.e., 12.9 ± 0.0-fold increase, *p* ≤ 0.001) but negligible effects for C/OA-NLC (i.e., 14.3 ± 0.1-fold increase). This suggests that C/LA-NLC is partially taken up via macropinocytosis. Blocking the caveolin-mediated pathway significantly (*p* ≤ 0.001) reduces particle uptake for both C/OA-NLC (10.5 ± 0.0-fold) and C/LA-NLC (10.4 ± 0.0-fold). This confirms that caveolin-mediated uptake is the predominant pathway, which implies a size-dependence. Clathrin-mediated uptake is predominant for particles smaller than 200 nm, while the caveolin-mediated pathway enables the uptake of particles with a size of up to 500 nm.^58^ This is in accordance with previous results by Jeitler et al.^35^ and Tetyczka et al.^59^ The pronounced cell uptake after 2 h also confirms the caveolin-mediated endocytosis, as this pathway requires more time than the clathrin-mediated pathway.^60^ Macropinocytosis is also independent of the size and shape of the particles.^61^ The colocalization studies show that the nanoparticles are primarily located near the ER and near the mitochondria, and no localization is observed in the lysosomes (Figure 5). This also agrees with the proposed pathway.^62^

## Conclusions

In summary, a preparation protocol for Compritol-based NLC with the three different liquid lipids (MCT, OA, and LA) was optimized to yield homogeneous particle size distributions. The particle size distributions and shapes of NLC were verified with LD and DLS experiments. ζ-Potentials were measured to assess and predict the stability of the colloidal dispersions. The thermodynamic properties of the NLC, as well as the binary and ternary mixtures of their individual components, were characterized with DSC to determine the miscibility of the components. All three types of NLC exhibit very similar properties in terms of the size distribution, shape, and overall composition. The coarse-grained simulations show that the three liquid lipids lead to different compositions of the NLC surface.

As shown by the simulations and the DSC data, the fully saturated liquid lipid MCT mixes with the solid lipid rather than with the stabilizer. As a result, no liquid lipid is present on the NLC surface, which prevents fusion with the cell membrane and thus the uptake into the cell. In contrast, unsaturated liquid lipids show better miscibility with the stabilizer, which increases as the number of double bonds increases. As a result, the NLC is covered by a lipid-stabilizer mixture on the surface, which enables the liquid lipid to come into contact with the cell membrane, leading to increased cell uptake. However, the increased fusogenicity can also lead to colloidal instabilities (i.e., recoalescence). Thus, cell uptake depends on the composition and structure of the NLC surface, which is a result of the balance of interactions between the individual components of the NLC. In addition to interactions between the matrix components, it is crucial to consider how these interactions are influenced by the drug candidate to be encapsulated. It is likely that the structural arrangement of the carrier will be affected; accordingly, it is expected that this will not only alter the manufacturing parameters but also have an impact on characteristics such as morphology, drug distribution within the particle itself, mechanical properties, carrier size, and physical stability. These changes may in turn affect key properties such as cellular uptake pathways, release behavior, and storage stability. Knowledge of the underlying molecular interactions of all NLC components is, therefore, essential for the fine-tuning of stable particle formulations with targeted cellular uptake mechanisms.

## Data Availability

The data that support the findings of this study are available within the main text and Supporting Information. Any other relevant data are available from the corresponding author upon request.

## Supporting Information Available

Additional experimental details, materials, methods, and results including statistical data analysis of DoE studies, characterization of NLC via RAMAN spectroscopy, methodology for molecular dynamics simulations, and cell interaction studies (PDF)

## Abbreviations

NLC: nanostructured lipid carriers
C: Compritol 888ATO
MCT: middle-chain triglycerides
OA: oleic acid
LA: linoleic acid
H_2_O_2_: hydrogen peroxide
EIPA: 5-(*N*-ethyl-*N*-isopropyl)amiloride
NEAA: nonessential amino acid
DoE: design of experiments
DMEM: Dulbecco’s modified Eagle’s medium
PBS: phosphate buffered saline
FBS: fetal bovine serum
HBSS: Hank’s balanced salt solution
EDTA: ethylenediaminetetraacetic acid
HPH: high-pressure homogenization
HSM: high-shear mixing
PdI: polydispersity Index
RI: refractive index
DLS: dynamic light scattering
ELS: electrophoretic light scattering
LD: laser diffraction
DSC: differential scanning calorimetry
cLSM: confocal laser scanning microscope
ER: endoplasmic reticulum
SD: standard deviation
*T*_m_: melting temperature;
