# Supplementary material for: On the Structure, Stability, and Cell Uptake of Nanostructured Lipid Carriers for Drug Delivery
Source: Mol Pharm. 2024 Jun 5;21(7):3674–83. doi: 10.1021/acs.molpharmaceut.4c00392 (PMC11220792; doi:10.1021/acs.molpharmaceut.4c00392)
Supplement: Supplementary file 1 — mp4c00392_si_001.pdf [file mp4c00392_si_001.pdf]

# On the structure, stability and cell uptake of nanostructured lipid carriers for drug delivery

*Ramona Jeitler [1,2], Christina Glader [1,2], Gerhard König [2,3], Jay Kaplan [2,4], Carolin Tetyczka [2], Johan Remmelgas [2], Marion Mußbacher [5], Eleonore Fröhlich [6], Eva Roblegg [1,2]**

[1] University of Graz, Institute of Pharmaceutical Sciences, Pharmaceutical Technology and Biopharmacy, 8010 Graz, Austria

[2] Research Center Pharmaceutical Engineering GmbH, 8010 Graz, Austria

[3] University of Portsmouth, Centre for Enzyme Innovation, School of Biological Sciences, Portsmouth PO1 2DY, United Kingdom

[4] University of Chicago, Pritzker School of Molecular Engineering, 60637 Chicago, Illinois, United States of America

[5] University of Graz, Institute of Pharmaceutical Sciences, Pharmacology and Toxicology, 8010 Graz, Austria

[6] Center for Medical Research, Medical University of Graz, Graz, Austria

# Supporting Information

## Materials

Compritol® 888 ATO (C; USP NF Name: Glyceryl dibehenate) was provided by Gattefossé (Saint Priest, France). It consists of mono-, di- and triesters of behenic acid (C22) with the diester fraction being the predominant. Oleic acid (OA) was provided by Croda GmbH (Nettetal, Germany). Medium-chain triglycerides (MCT) were purchased from Herba Chemosan Apotheker-AG (Vienna, Austria). Linoleic acid (LA), Tween® 80 (Tween 80), oil-red-o, hydrogen peroxide (H2O2), dynasore hydrate, chlorpromazine hydrochloride, genistein, 5-(N-Ethyl-N-isopropyl)amiloride (EIPA) and MEM non-essential amino acid solution (100 x; NEAA) were obtained from Sigma Aldrich (Munich, Germany). Human buccal TR146 cells from Imperial Cancer Research Technology (London, UK) were used for all cell culture experiments. Dulbecco's Modified Eagle's medium (DMEM), phosphate buffered saline (PBS; pH 7.4), fetal bovine serum (FBS), penicillin streptomycin (Penstrep), Hank's Balanced Salt Solution (HBSS), and 0.25% trypsin-ethylenediaminetetraacetic acid (trypsin-EDTA) were obtained from Gibco, Life Technologies Corporation (Painsley, UK). Dihydroethidium (DHE), Alexa Fluor 488 Phalloidin and Hoechst 33342 were purchased from Thermo Fisher Scientific (Vienna, Austria). HyClone (i.e., serum-free DMEM) was obtained from GE Healthcare Life Sciences (Logan, USA).

Ultrapurified water (i.e., Milli-Q®-water (MQ-water); Millipore SAS, Molsheim, France) was used for all experiments.

# Methods

## Characterization of NLC: Raman spectroscopy

Raman spectroscopy investigations were performed using a RamanStation 400F spectrometer (Perkin Elmer, Waltham, Massachusetts) equipped with a cooled charged-coupled (-50 ˚C) 256 x 1024 CCD detector and a 350 mW near-infrared 785 nm laser. The bulk materials and the dried NLC were placed on the sample copper holder. Subsequently, the laser was focused and SuperMacro point measurements were conducted using an exposure number of ten and an exposure time of 10 s each. Spectra in the range of 200-3250 $cm^{-1}$ with a resolution of 2 $cm^{-1}$ were acquired and samples were analyzed after the baseline correction and peak normalization using the software Spectragryph (F. Menges Spectragryph – spectroscopy software, version 1.2.16, 2022).

## Coarse-grained molecular dynamics simulations to simulate the NLC structure

### Methodology for molecular dynamics simulations

The Martini Force Field 2.0 [1,2] with polarizable water was used to model all system components. Structures and standard parameters for the solid lipid C, the liquid lipids MCT, OA and LA were adapted from Marrink et al. [1]. The stabilizer, Tween 80, used the 4-H Tween 80 structure as described by Luz et al. [3].

Two sets of simulations were run. The first set, which consisted of the pure components in a cubic box with an initial volume of 42.4 x 42.4 x 42.4 nm, was used to calculate component densities and to validate the coarse-grained structures.

The second set simulated a slab of the NLC, thus employing the spherical symmetry of the NLC to reduce the computational costs. The initial volume of the slab was 42.4 × 42.4 × 260 nm, which was used to analyze the density profiles of the NLC consisting of the three different liquid lipids (MCT, OA, and LA). Phase profiles along the direction perpendicular to the interface were obtained using the species number density as a function of distance along the slab to investigate the concentrations of the solid lipid, the liquid lipid, the surfactant, and the solvent phases. The initial rectangular nanoparticle slab simulation box had an extended side length of 220 nm, equal to the average experimental diameter of one nanoparticle in order to accurately capture layer widths. The short box side lengths were set to 42.4 nm to reduce the potential error between the arc (full NLC sphere) and line (rectangular slab) to below 1%. A 20 nm layer of polarizable water was included on either side of the NLC slab. Periodic boundary conditions were used. The box contained a central layer of solid lipids bounded on either side by a layer of liquid lipids, followed by the stabilizer layer. The number of molecules in each layer matched the experimental molar ratios of the NLC. The exact species numbers are listed in Supporting Table 1. The layer width was determined based on the density and molar ratios of each component. The systems were generated using the software package Packmol [4].

**Supporting Table 1** Species number for each set of slab simulations

| Simulation | Polarizable water | Tween 80 | Liquid lipid (chain length composition) | | Solid lipid (C) | |
|---|---|---|---|---|---|---|
| MCT | 8000 | 332 | MCT (8:8) | 244 | monoglyceride | 756 |
| | | | MCT (8:10) | 266 | diglyceride | 1078 |
| | | | MCT (10:10) | 188 | triglyceride | 458 |
| OA or LA | 8000 | 344 | OA/LA | 722 | monoglyceride | 783 |
| | | | | | diglyceride | 1115 |
| | | | | | triglyceride | 475 |

## Cell culture: Cell interaction studies

TR146 cells were seeded in 96-well plates (Greiner Bio-One GmbH, Frickenhausen, Germany) using a seeding density of 2 x $10^4$ cells/well and cultured for 72 h. After a washing step with PBS, the cells were incubated with NLC diluted in serum-free DMEM at different concentrations (i.e., 100 – 1000 µg/ml) for 4 h (n=6). For the determination of the cell viability, a CellTiter 96 Aqueous Non-Radioactive Cell Proliferation Assay (MTS, Promega Corporation, Madison, USA) was used according to the manufacturer's instructions. After NLC incubation, cells were washed and then incubated with MTS reagent for 3 h. Absorbance was measured at 490 nm using a microplate reader (CLARIOstar$^{Plus}$, BMG LABTECH, Ortenberg, Germany). Untreated blank-corrected wells representing 100% cell viability were used as a control. The release of lactate dehydrogenase (LDH) was determined after 4 h incubation with the respective NLC formulations (i.e., 100 – 1000 µg/ml) using a CytoTox-ONE Homogeneous Membrane Integrity Assay (Promega) according to manufacturer's instructions. The fluorescence intensity was investigated at an

excitation wavelength of 560 nm and an emission wavelength of 590 nm using a microplate reader (CLARIOstar$^{Plus}$, BMG LABTECH). Cells treated with 2% of a lysis solution were used as controls to represent 100% LDH release. All data were corrected for blank values.

For investigating reactive oxygen species (ROS) release after incubation with the NLC-formulations, TR146 cells were seeded in 96-well plates (Nunc™ MicroWell™ 96-Well Optical-Bottom Plates with Polymer Base; Thermo Fisher Scientific) with a seeding density of 6 x $10^4$ cells/well and cultured for 24 h. After washing with PBS, cells were incubated with NLC formulations in different concentrations (i.e., 100 – 1000 µg/ml; diluted with serum-free DMEM) containing 10 µM DHE for 4 h (n=6). Untreated wells served as a control. The fluorescence was investigated at an excitation wavelength of 544 nm and an emission wavelength of 612 nm using the CLARIOstar$^{Plus}$ (BMG LABTECH).

## Statistical analysis

If not otherwise stated, experiments were performed in triplicate and results were presented as mean values ± standard deviation (SD). Statistical analyses were conducted via Student's t tests. Differences were considered to be significant at a level of $p \leq 0.05$ (*), $p \leq 0.01$ (**) and $p \leq 0.001$ (***).

# Results

## Preparation of NLC with defined characteristics using DoE

As a first step, production conditions were screened to yield uniform size distributions for all three kinds of NLC to avoid artefacts in the cell uptake experiments. No outliers (i.e., ± 4 SD) are detected in the normal probability plots, and all experiments exhibit a linear distribution, which allows the creation of a linear model to guide the search for suitable parameters (see Supporting Fig. 1).

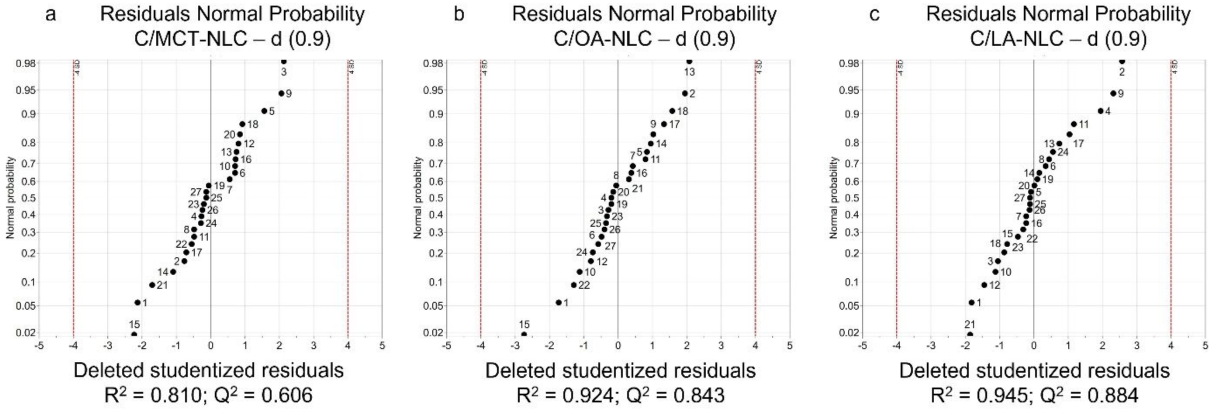


**Supporting Figure 1** Residual normal probability plots of a) C/MCT-, b) C/OA- and c) C/LA-NLC created with the Modde® software. No outliers (i.e., ± 4 SD) are detected in the normal probability plots, and all experiments exhibit a linear distribution, regardless of the formulation tested. The $R^2$ values indicate a high fit between the variability observed and the regression model and the $Q^2$ values describe a precision and reliability of future prediction.

The established models exhibit $R^2$ values of 0.810 (C/MCT-NLC), 0.924 (C/OA-NLC), and 0.945 (C/LA-NLC), indicating a very good fit between the observed variability and the regression

model (see Supporting Fig. 1 a-c). Moreover, high $Q^2$ values of 0.606 (C/MCT-NLC), 0.843 (C/OA-NLC), and 0.884 (C/LA-NLC) indicate high predictive precision. Further center point experiments indicate high reproducibility (i.e., 99.9%) and robustness of the model for all formulations. The coefficient plots of the model reveal that the stabilizer concentration (Stab) and its quadratic effects (Stab*Stab) are the most significant factors affecting the particle size, independent of the matrix composition (see Supporting Fig. 2 a-c). This is in accordance with previous findings in the literature [5,6]. Higher stabilizer concentrations are associated with smaller particle sizes, but due to the opposite signs of the linear and quadratic terms only until a saturation point. Beyond this point, no further size reduction is observed [7,8]. For C/MCT formulations, besides number of cycles (Cycle), the solid-to-liquid lipid ratios (Ratio) significantly affect the particle size: a 9:1 (w:w) ratio leads to significantly smaller particles compared to 7:3 (w:w) mixtures. Furthermore, a strong interdependence between pressure and stabilizer (Pres*Stab) is found (see Supporting Fig. 2 a). This correlation is also evident in C/OA and C/LA formulations. Although a higher number of cycles and a higher pressure generally lead to a reduction in particle size, over-processing poses the risk of increasing particle size. Additional energy cannot be used for further lipid dispersion when the maximum dispersity is reached. It merely accelerates the particle velocity, creating enough kinetic energy to overcome the surfactant stabilization. This leads to aggregation and an increase in mean size, which is the case for C/OA and C/LA formulations. For C/OA and C/LA formulations, also the interaction between stabilizer and the lipid ratio is size-affecting, which is influenced by factors such as HLB values, material miscibilities, and molecular geometry (see Supporting Fig. 2 b, c) [7,9,10].

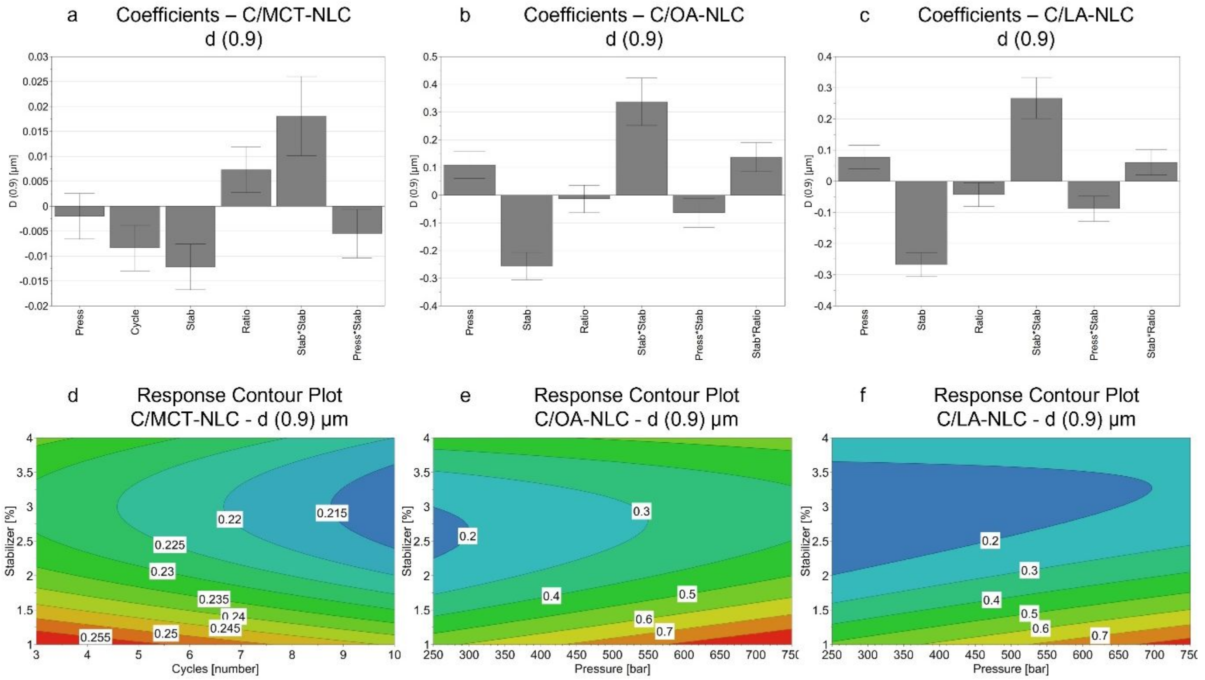


**Supporting Figure 2** Coefficient plots and response contour plots for C/MCT-NLC (a, d) at a fixed homogenization pressure (i.e., 500 bar), C/OA-NLC (b, e) and C/LA-NLC (c, f) at a fixed number of cycles (i.e., eight) using d (0.9)-values as response [µm]. The solid-to-liquid lipid ratio (i.e., 9:1 (w:w)) was fixed for all formulations. Statistical analysis was performed with the Modde® software. Abbreviations: HPH pressure (Press) [bar], number of HPH cycles (Cycle), stabilizer concentration (Stab) [% (w/w)], solid-to-liquid lipid ratio (Ratio) [w:w], quadratic interactions (e.g., Stab*Stab), interaction between two parameters (e.g., Press*Stab).

To provide a graphical interpretation of interactions between process variables and formulation composition, 2D contour plots of the two dominant dimensions of the regression models were generated (see Supporting Fig. 2 d-f). To investigate the cell uptake process as a function of matrix

composition, particularly with respect to the contribution of the liquid lipid, it is crucial to maintain a uniform particle size (i.e., d (0.9) = 200-300 nm) and a narrow size distribution across all formulations [11]. Therefore, a fixed solid-to-liquid lipid ratio of 9:1 (w:w) is chosen, and the most influential parameters are plotted against each other (see Supporting Fig. 2 d for C/MCT-formulations and Supporting Fig. 2 e and f for C/OA and C/LA formulations).

The contour plot for C/MCT-NLC indicates that a high number of cycles is required to obtain smaller particles. Conversely, for C/OA and C/LA mixtures, over-processing at too high process pressures results in larger particle sizes. To subject all three formulations to similar mechanical stresses, eight cycles at 500 bar are chosen for all mixtures. Under these conditions, the contour diagrams show that a stabilizer concentration of approximately 2-3.5% (w/w) is necessary to attain the desired NLC particle sizes. Consequently, a fixed stabilizer concentration of 2.5% (w/w) is used for all NLC mixtures to ensure similar size distributions in the cell uptake experiments of the three NLC types. This concentration is also chosen to mitigate the risk of cytotoxic effects of the stabilizer [9,12].

## Characterization of NLC

### Storage stability

LD measurements (see Supporting Fig. 3) coincide with the DLS findings, showing d (0.9) values of 212 ± 0 nm for C/MCT, 219 ± 1 nm for C/OA, and 227 ± 2nm for C/LA-NLC at day zero. This corresponds to size differences of about 7% between the three NLC types. Notably, the d (0.99) values confirm the absence of large particles in the C/MCT formulation (i.e.,

d (0.99) 280 ± 0 nm). Conversely, small fractions of larger particles are observed in C/OA-NLC (i.e., d (0.99) 750 ± 0 nm) and C/LA-NLC (i.e., d (0.99) 910 ± 0 nm). However, as the d (0.95) values remain below 280 ± 0 nm for all formulations, it can be concluded that less than 5% of the particles form agglomerates. Stability studies performed over 28 days of storage at room temperature (C/MCT) or at 5 ± 3 ˚C (C/OA and C/LA) show that d (0.9) values for C/MCT and C/OA formulations remain stable (i.e., 216 ± 0 nm for C/MCT and 225 ± 0 nm for C/OA). In contrast, C/LA formulations gel after two weeks, revealing a notable change in physical properties.

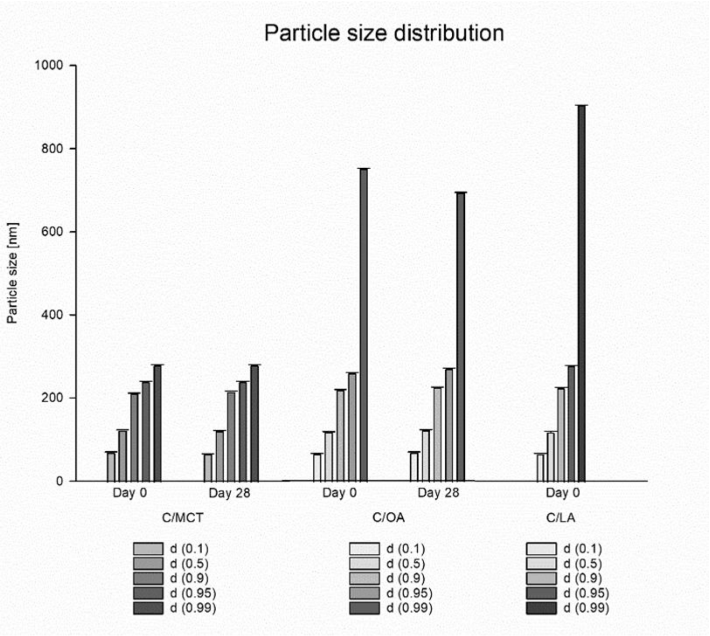


**Supporting Figure 3** Particle sizes [nm] measured via LD of C/MCT-, C/OA- and C/LA-NLC expressed as d (0.1), d (0.5), d (0.9), d (0.95) and d (0.99) values on the day of production (Day 0) and after 28 days (Day 28) of storage. All values are presented as mean values ± SD.

Miscibility studies using DSC and RAMAN

The bulk material of the solid lipid shows a melting peak at $T_m$=75.3 ± 0.5 ˚C (onset at 70.7 ± 0.2 ˚C). The DSC curves of the liquid lipids reveal melting peaks at $T_m$=-3.50 ± 0.0 ˚C (onset at -12.8 ± 0.7 ˚C) for MCT, $T_m$=14.7 ± 0.1 ˚C (onset at 8.3 ± 0.1 ˚C) for OA and $T_m$=-0.85 ± 0.6 ˚C (onset at 6.9 ± 0.4 ˚C) for LA (see Supporting Fig. 4).

Recrystallization of the bulk solid lipid occurs at the recrystallization temperature ($T_r$) of 65.9 ± 0.6 ˚C (onset at 68.1 ± 0.1 ˚C) and shifts to lower temperatures for NLC formulations (i.e., $T_r$=64.3 ± 0.3 ˚C with an onset at 67.2 ± 0.1 ˚C for C/MCT, $T_r$=65.2 ± 0.1 ˚C with an onset at 66.8 ± 0.0 ˚C for C/OA and $T_r$=63.7 ± 0.8 ˚C with an onset at 66.7 ± 0.1 ˚C for C/LA). These results suggest that the NLC are present in the crystalline form.

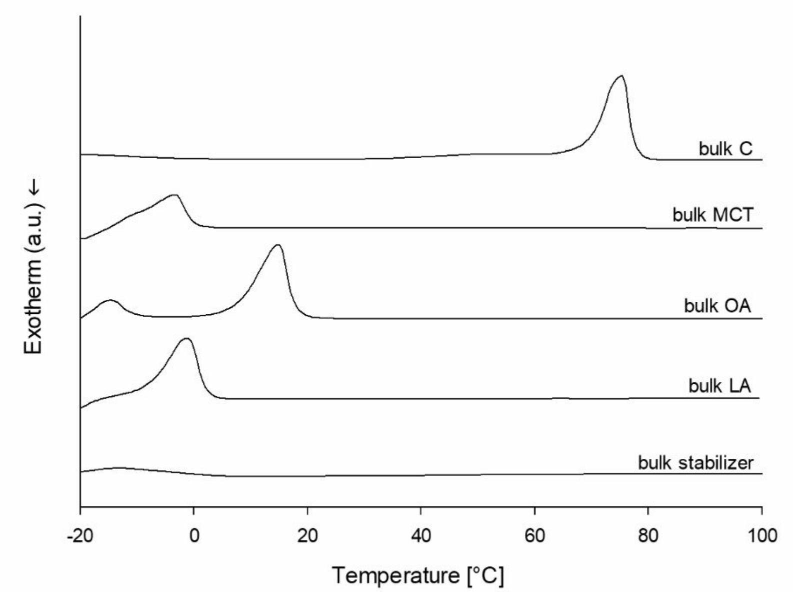


**Supporting Figure 4** DSC thermograms of bulk materials C, MCT, OA, LA and the stabilizer (Tween 80).

Raman spectroscopy investigations were conducted on all bulk materials revealing a distinct Raman shift at 1438 $cm^{-1}$, indicative of $CH_2$ scissoring vibrations [13] (see Supporting Fig. 5). In comparison to the solid lipid, the liquid lipids exhibit broader peaks due to their less ordered crystalline structure [14]. Notably, the solid lipid displays more prominent peaks at 1062 $cm^{-1}$ and 1128 $cm^{-1}$, corresponding to C-C asymmetric and C-C symmetric stretching vibrations, respectively [15]. Raman shifts at 1298 $cm^{-1}$ (solid lipid) and 1304 $cm^{-1}$ (liquid lipids) correspond to $CH_2$ groups [16].

A distinctive carbonyl peak of ester bonds at 1736 $cm^{-1}$ is observed for MCT [17], while typical peaks associated with the unsaturated fatty acids (i.e., OA and LA) appear at 1265 $cm^{-1}$ (=C-H stretching mode) and 1655 $cm^{-1}$ (C=C stretching vibrations). The ratio of the peaks at 1655 $cm^{-1}$ and 1444 $cm^{-1}$ provides insights into the degree of unsaturation, with LA exhibiting a higher ratio compared to OA (i.e., 1.54 and 0.99), signifying a greater degree of unsaturation [18].

Raman spectroscopy studies of the NLC reveal shifts comparable to those observed in the bulk solid lipid. Notably, the characteristic MCT peak at 1736 $cm^{-1}$ is absent in C/MCT NLC. In contrast, the Raman shifts at 1655 $cm^{-1}$ in C/OA and C/LA NLC are attributed to the liquid lipid component. None of the three formulations exhibit peaks at 1304 $cm^{-1}$ or broadening of the peak at 1298 $cm^{-1}$, indicating the absence of free liquid lipid [19]. This also suggests at least partial miscibility of C and MCT as the peaks at 1304 $cm^{-1}$ and at 1736 $cm^{-1}$ in C/MCT cannot be detected and the typical peaks for OA and LA (i.e., 1655 $cm^{-1}$) are also present in the NLC formulation [19]. Furthermore, no significant frequency or intensity shifts are observed, which indicates that no polymorphic transitions occur during the production of NLC [20].

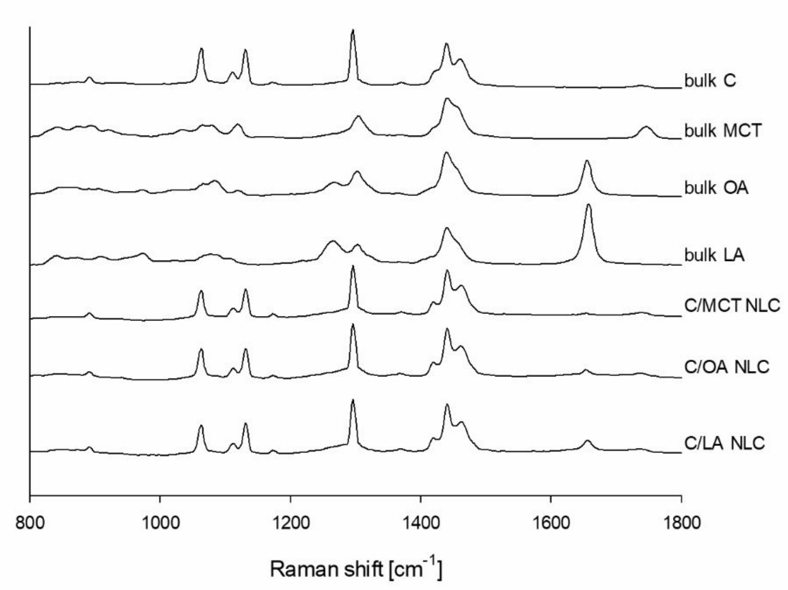


**Supporting Figure 5** Raman spectra of the bulk materials C, MCT, OA and LA and NLC formulations (i.e., C/MCT-NLC, C/OA-NLC and C/LA-NLC).

## Coarse-grained molecular dynamics simulations to simulate the NLC structure

### Structural and thermodynamic properties

To compute the slab density profiles of each component, the GROMACS density function was used. The results were averaged over four production simulations, with each simulation being sampled from both ends of the slab. The simulations were visualized using VMD software program [21].

$$N(d) = \mathrm{B} - \mathrm{A} \cdot \mathrm{ERF}\left(\frac{d-D}{\sqrt{2}\cdot W}\right) \qquad [1]$$

The density profile at a phase interfacial boundary can be fitted with a hyperbolic error function as is standard practice described in Benayad et al [22]. The density profiles were fitted to equation 1

using the Python SciPy package [23]. In equation 1, *N(d)* is the number density of a species as a function of the distance from the center of the slab, *d*, *ERF* is the error function, and *A*, *B*, *D*, *W* are fitted parameters. The parameters *A* and *B* together determine the dense and dilute phase concentrations, *D* determines the horizontal translation of the phase boundary, and *W* determines the sharpness of the boundary [22]. Standard errors are calculated from the covariance matrix of the nonlinear fit.

$$c_{dense} = B + A \quad [2]$$

$$c_{dilute} = B - A \quad [3]$$

$$\Delta G = k_b \mathrm{T} \cdot \ln\left(\frac{c_{dense}}{c_{dilute}}\right) \quad [4]$$

For defined density plateaus, equations 2 and 3 give the concentration of a component in the dense, $c_{dense}$, and dilute, $c_{dilute}$, phases respectively. Equation 4, in which $k_b$ is Boltzmann's constant and *T* is the temperature (K), approximates the Gibbs transfer free energy, defined as the energy required to bring a molecule from the dilute to the dense phase.

### Gibbs transfer free energy

Supporting Figure 6 shows that the Gibbs transfer free energy of the solid lipid moving between the solid lipid bulk phase and liquid lipid bulk phase increases with increasing degree of unsaturation. This indicates a lower miscibility between the solid and the liquid lipid for OA and LA, which also coincides with the DSC data.

Conversely, the opposite occurs between the liquid lipid and the stabilizer. As the degree of unsaturation increases in the liquid lipid, the phase boundary to the stabilizer becomes less well-

defined, and the Gibbs transfer free energy decreases. This means that unsaturated lipids are more miscible with the stabilizer. Accordingly, the simulations show that the lipid layers do not mix with the surfactant effectively in the case of the saturated MCT. The hydrophobicity of the MCT surface promotes the coverage of newly formed NLC interfaces by the liquid lipid and the stabilizer's hydrophobic component. The coverage of the hydrophobic surface is independent of the applied homogenization pressure, which is in accordance with the results of DoE studies. Therefore, a sufficient stabilizer concentration is essential for achieving smaller particle sizes.

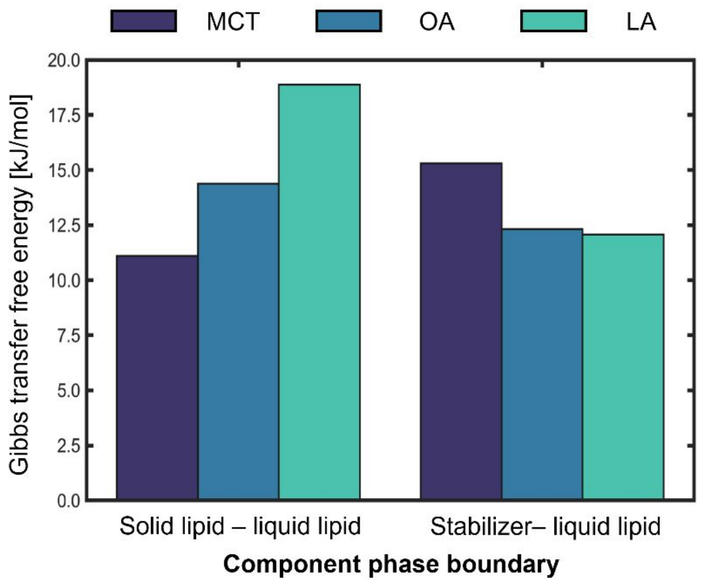


**Supporting Figure 6** Miscibilities of the liquid lipid with the solid lipid or the stabilizer based on the Gibbs transfer free energy (n=8; based on four simulations where both ends of the slab were evaluated). The left side shows the Gibbs transfer free energy of a solid lipid molecule from the dense phase (solid lipid bulk) into the dilute phase (liquid lipid bulk). The right side shows the Gibbs transfer free energy of a stabilizer molecule from the dense phase (stabilizer bulk) into the

dilute phase (liquid lipid bulk). The concentrations in the dense and dilute phase were obtained by fitting the phase profiles to equation 1 and the free energies were calculated from equation 2.

### Radial distribution functions

The interactions of the main components of the NLC surface are further characterized by radial distribution functions from the simulations (see Supporting Fig. 7). The radial distribution functions indicate the probability of finding the two shown interaction partners at a certain distance, and high probabilities at short distances are an indicator of attractive interactions. The intensity of the interactions of the stabilizer Tween 80 with other Tween 80 molecules decreases with increasing unsaturation of the liquid lipid (MCT<OA<LA). At the same time, the interactions of the liquid lipid with the hydrophobic tail of the stabilizer increase with increasing unsaturation of the liquid lipid. This means that more stabilizer is bound to the unsaturated liquid lipids. The hydrophobic tail of the stabilizer favors interactions with the liquid lipid, while the hydrophilic tails of the stabilizer mostly remain in contact with water. Both kinds of interactions can be satisfied at the same time at the surface between the nanoparticle and the aqueous solution. The binding to the stabilizer is stronger in LA than in OA or MCT. With increasing desaturation, the interactions between the solid and the liquid lipid decrease. For NLC with LA, the interactions of the liquid lipid with the solid lipid are very weak, which might explain the low stability.

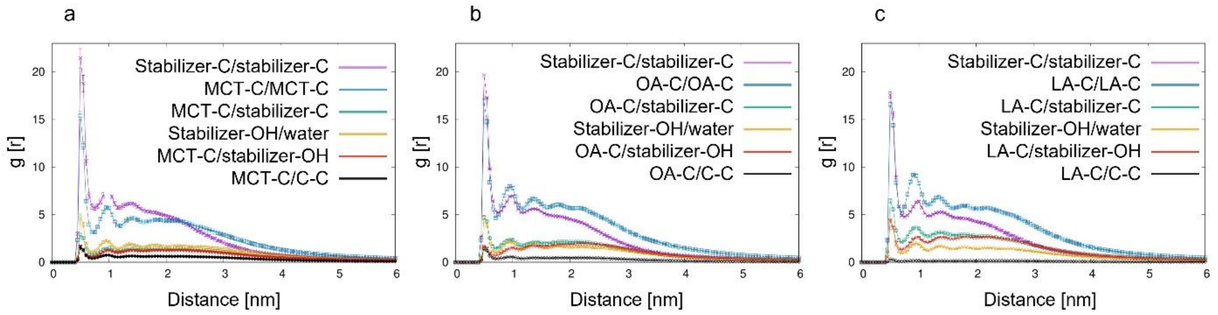


**Supporting Figure 7** Radial distribution functions of two interaction partners at a certain distance throughout the simulation for C/MCT-NLC (a), C/OA-NLC (b) and C/LA-NLC (c), where -C represents the second bead of the hydrophobic tail of the liquid lipids and the stabilizer. For Tween 80 (stabilizer), OA, and LA, this is also the bead that contains the double bond. (purple) The coarse-grained bead of the stabilizer that contains the double bound with beads of the same type in other stabilizer molecules (Stabilizer-C/stabilizer-C); (blue) The second bead of the hydrophobic tail of MCT with beads of the same type in other MCT molecules (MCT-C/MCT-C), (A) and OA/LA that contain the double-bond with beads of the same type in other OA/LA molecules (OA-C/OA-C, LA-C/LA-C); (green) The second bead of the hydrophobic tail of MCT (A) and OA/LA (B, C) that contain the double-bond with the bead of the hydrophobic tail of the stabilizer that contains a double bond (MCT/OA/LA-C/stabilizer-C); (yellow) The terminal bead with the hydroxyl group of the hydrophilic tails of the stabilizer and water (Stabilizer-OH/water); (orange) The second bead of the hydrophobic tail of MCT (A) and OA/LA (B, C) that contain the double-bond with the terminal bead that contains the hydroxyl group of the hydrophilic tails of the stabilizer (MCT/OA/LA-C/stabilizer-OH); (black) The second bead of the hydrophobic tail of MCT (A) and OA/LA (B, C) that contains the double-bond with the second bead of the hydrophobic tail of C.

## Cell culture: Cell interaction and uptake studies

Cell uptake studies were performed on the buccal epithelial model cell line TR146. The compatibility tests indicate that in TR146 cells, both C/MCT- and C/OA-NLC show no significant cytotoxic effects, with cell viability ranging from 95% to 99% regardless of the particle concentration (see Supporting Fig. 8). However, C/LA-NLC exhibit a dose-dependent decrease in cell viability, with a significant decrease ($p \leq 0.01$) at higher concentrations (750 and 1000 µg/ml). Nevertheless, cell viability remains above 70% suggesting that no damage to the mitochondria occurred [24]. LDH release does not increase with the C/MCT- and C/OA-NLC formulations at the concentrations tested, indicating no effect on cell membrane integrity. In contrast, C/LA-NLC show a significant ($p \leq 0.01$) LDH release of up to 18% at higher concentrations. As LDH release remains below the threshold of 30% for all formulations, a minimal and thus permissible damage to the cell membrane within the tested range can be assumed [24] (see Supporting Fig. 8).

Exposure of TR146 cells to NLC (C/MCT, C/OA, and C/LA) at varying concentrations results in ROS production measured with DHE (see Supporting Fig. 9). Lower concentrations (100 – 500 µg/ml) of C/MCT and C/LA NLC show no significant difference in ROS levels compared to the control. However, higher concentrations (i.e., 750 and 1000 µg/ml) lead to a 1.3- and 1.4-fold increase in ROS production for C/MCT-NLC ($p \leq 0.001$) and a 1.3-fold increase for C/LA-NLC ($p \leq 0.01$). In contrast, all concentrations of C/OA-NLC exhibit significantly higher ROS levels, with up to a 1.6-fold increase ($p \leq 0.001$). As these effects do not severely impact TR146 cell function in toxicity studies, subsequent cellular uptake investigations are conducted at concentrations of 500 and 750 µg/ml.

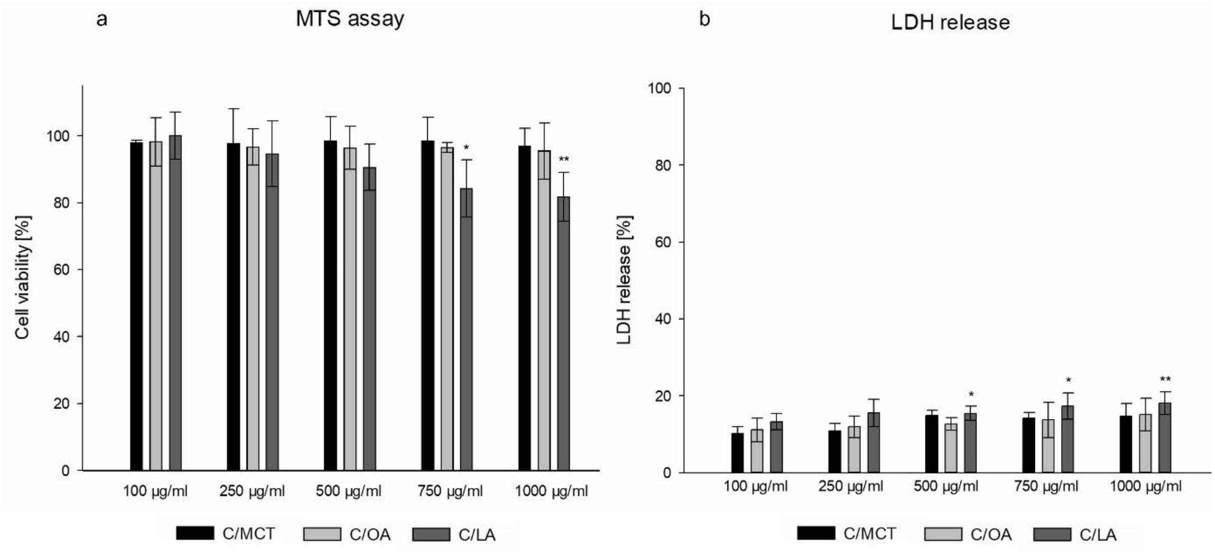


**Supporting Figure 8** Cell viability a) and LDH release b) of TR 146 cells treated with 100 – 1000 µg/ml C/MCT-, C/OA- and C/LA-NLC. The percentages given (% ± SD) refer to the corresponding controls. Student's t-test was used for statistical analysis. Significant differences compared to the control are marked as * and ** which correspond to a p value ≤ 0.05 and ≤ 0.01.

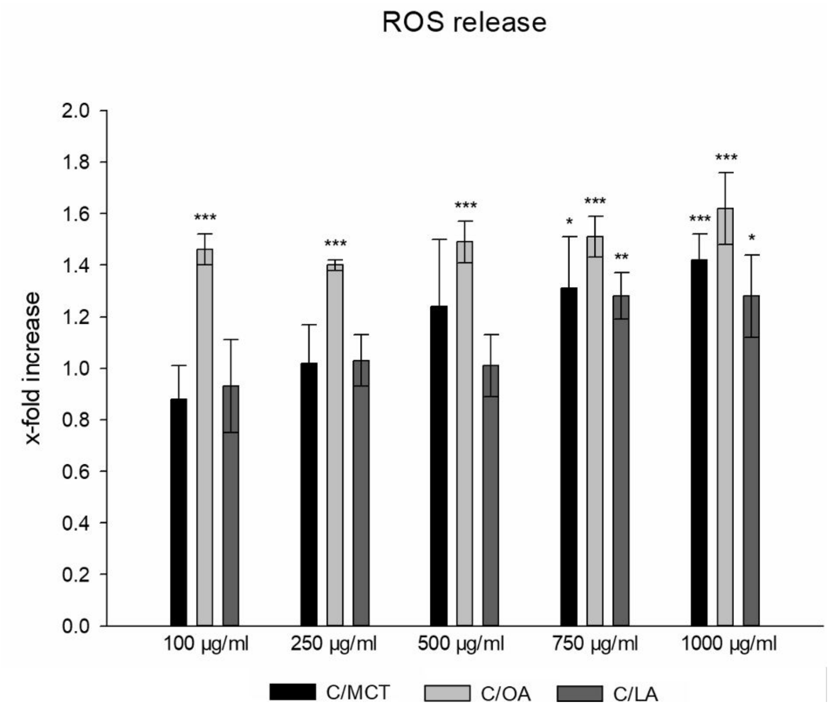


**Supporting Figure 9** Generation of ROS after treatment with NLC (i.e., C/MCT, C/OA and C/LA) in a concentration dependent manner (i.e., 100 – 1000 µg/ml) compared to untreated TR146 cells. Student's t-test was used for statistical analysis. Significant differences compared to the control are marked as *, ** and *** which corresponds to a p value of ≤ 0.05, ≤ 0.01 and ≤ 0.001.

AUTHOR INFORMATION

*** Corresponding Author**

Univ.-Prof. Dr. Eva Roblegg

University of Graz

Institute of Pharmaceutical Sciences,

Department Pharmaceutical Technology and Biopharmacy

Universitätsplatz 1, A-8010 Graz, Austria

Phone: +43 316 380-8888

Fax: +43 316 380-9100

Email: eva.roblegg@uni-graz.at

eva.roblegg@uni-graz.at

**Author Contributions**

The manuscript was written through contributions of all authors. All authors have given approval to the final version of the manuscript. R.J., C.G., G.K. and E.R. conceptualized the work, R.J., C.G. and C.T. produced the experimental data, G.K. and J.K. performed the molecular simulations

under the supervision of G.K. and J.R., E.F. and M.M. supported the SFC and cLSM studies and data interpretation, R.J., C.G., G.K., J.K. and E.R. interpreted the experimental and simulation data and wrote the manuscript. All authors have read and commented on the manuscript.

**Funding Sources**

The Research Center Pharmaceutical Engineering (RCPE) is funded within the framework of COMET - Competence Centers for Excellent Technologies by BMK, BMDW, Land Steiermark and SFG. The COMET program is managed by the FFG. This research did not receive any specific grant from funding agencies in the public, commercial or not-for-profit sectors.

**Acknowledgment**

The authors acknowledge the financial support by the University of Graz.

**Abbreviations**

NLC, nanostructured lipid carriers; C, Compritol® 888ATO; MCT, middle-chain triglycerides; OA, oleic acid; LA, linoleic acid; $H_2O_2$, hydrogen peroxide; EIPA, 5-(N-Ethyl-N-isopropyl)amiloride; NEAA, non-essential amino acid; DoE, Design of Experiments; DMEM, Dulbecco's Modified Eagle's medium; PBS, phosphate buffered saline; FBS, fetal bovine serum; HBSS, Hank's Balanced Salt Solution; EDTA, ethylenediaminetetraacetic acid; LDH, lactate dehydrogenase; ROS, reactive oxygen species; HPH, high-pressure homogenization; HSM, high-shear mixing; PdI, Polydispersity Index; RI, refractive index; DLS, Dynamic Light

Scattering; ELS, Electrophoretic Light Scattering; LD, Laser Diffraction; DSC, Differential Scanning Calorimetry; cLSM, confocal laser scanning microscope; ER, endoplasmic reticulum; SD, standard deviation; $T_m$, melting temperature;

## Competing interests

The authors declare no competing interests.

## Data Availability

The data that support the findings of this study are available within the main text and Supporting Information. Any other relevant data are available from the corresponding author upon request.

## References


(1) Marrink, S. J.; Risselada, H. J.; Yefimov, S.; Tieleman, D. P.; de Vries, A. H. The MARTINI Force Field: Coarse Grained Model for Biomolecular Simulations. *J Phys Chem B* **2007**, *111* (27), 7812–7824. https://doi.org/10.1021/jp071097f.

(2) Yesylevskyy, S. O.; Schäfer, L. V.; Sengupta, D.; Marrink, S. J. Polarizable Water Model for the Coarse-Grained MARTINI Force Field. *PLoS Comput Biol* **2010**, *6* (6), e1000810. https://doi.org/10.1371/journal.pcbi.1000810.

(3) Luz, A. M.; Barbosa, G.; Manske, C.; Tavares, F. W. Tween-80 on Water/Oil Interface: Structure and Interfacial Tension by Molecular Dynamics Simulations. *Langmuir* **2023**, *39* (9), 3255–3265. https://doi.org/10.1021/acs.langmuir.2c03001.

(4) Martínez, L.; Andrade, R.; Birgin, E. G.; Martínez, J. M. PACKMOL: A Package for Building Initial Configurations for Molecular Dynamics Simulations. *J Comput Chem* **2009**, *30* (13), 2157–2164. https://doi.org/10.1002/jcc.21224.

(5) Shegokar, R.; Singh, K. K.; Müller, R. H. Production & Stability of Stavudine Solid Lipid Nanoparticles - from Lab to Industrial Scale. *Int J Pharm* **2011**, *416* (2), 461–470. https://doi.org/10.1016/j.ijpharm.2010.08.014.

(6) Ghanem, H. A.; Nasr, A. M.; Hassan, T. H.; Elkhoudary, M. M.; Alshaman, R.; Alattar, A.; Gad, S. Comprehensive Study of Atorvastatin Nanostructured Lipid Carriers through Multivariate Conceptualization and Optimization. *Pharmaceutics* **2021**, *13* (2), 178. https://doi.org/10.3390/pharmaceutics13020178.

(7) McClements, D. J.; Jafari, S. M. Improving Emulsion Formation, Stability and Performance Using Mixed Emulsifiers: A Review. *Adv Colloid Interface Sci* **2018**, *251*, 55–79. https://doi.org/10.1016/j.cis.2017.12.001.

(8) Mehnert, W.; Mäder, K. Solid Lipid Nanoparticles Production, Characterization and Applications. *Adv Drug Deliv Rev* **2001**, *47* (2–3), 165–196. https://doi.org/10.1016/S0169-409X(01)00105-3.

(9) Lüdtke, L. F.; Stahl, A. M.; Grimaldi, R.; Forte, M. B. S.; Gigante, L. M.; Ribeiro, A. P. B. Optimization of High Pressure Homogenization Conditions to Produce Nanostructured Lipid Carriers Using Natural and Synthetic Emulsifiers. *Food Res Int* **2022**, *160*, 111746. https://doi.org/10.1016/j.foodres.2022.111746.

(10) Peng, J.; Dong, W.; Li, L.; Xu, J.; Jin, D.; Xia, X.; Liu, Y. Effect of High-Pressure Homogenization Preparation on Mean Globule Size and Large-Diameter Tail of Oil-in-Water Injectable Emulsions. *J Food Drug Anal* **2015**, *23* (4), 828–835. https://doi.org/10.1016/j.jfda.2015.04.004.

(11) Han, J. Y.; La Fiandra, J. N.; DeVoe, D. L. Microfluidic Vortex Focusing for High Throughput Synthesis of Size-Tunable Liposomes. *Nat Commun* **2022**, *13* (1), 6997. https://doi.org/10.1038/s41467-022-34750-3.

(12) Tetyczka, C.; Griesbacher, M.; Absenger-Novak, M.; Fröhlich, E.; Roblegg, E. Development of Nanostructured Lipid Carriers for Intraoral Delivery of Domperidone. *Int J Pharm* **2017**, *526* (1–2), 188–198. https://doi.org/10.1016/j.ijpharm.2017.04.076.

(13) Numata, Y.; Kobayashi, H.; Oonami, N.; Kasai, Y.; Tanaka, H. Simultaneous Determination of Oleic and Elaidic Acids in Their Mixed Solution by Raman Spectroscopy. *J Mol Struct* **2019**, *1185*, 200–204. https://doi.org/10.1016/j.molstruc.2019.02.110.

(14) Castro, S. R.; Ribeiro, L. N. M.; Breitkreitz, M. C.; Guilherme, V. A.; Rodrigues da Silva, G. H.; Mitsutake, H.; Alcântara, A. C. S.; Yokaichiya, F.; Franco, M. K. K. D.; Clemens, D.; Kent, B.; Lancellotti, M.; de Araújo, D. R.; de Paula, E. A Pre-Formulation Study of Tetracaine Loaded in Optimized Nanostructured Lipid Carriers. *Sci Rep* **2021**, *11* (1), 21463. https://doi.org/10.1038/s41598-021-99743-6.

(15) Saupe, A.; Gordon, K. C.; Rades, T. Structural Investigations on Nanoemulsions, Solid Lipid Nanoparticles and Nanostructured Lipid Carriers by Cryo-Field Emission Scanning Electron Microscopy and Raman Spectroscopy. *Int J Pharm* **2006**, *314* (1), 56–62. https://doi.org/10.1016/j.ijpharm.2006.01.022.

(16) Rodrigues da Silva, G. H.; Ribeiro, L. N. M.; Mitsutake, H.; Guilherme, V. A.; Castro, S. R.; Poppi, R. J.; Breitkreitz, M. C.; de Paula, E. Optimised NLC: A Nanotechnological Approach to Improve the Anaesthetic Effect of Bupivacaine. *Int J Pharm* **2017**, *529* (1–2), 253–263. https://doi.org/10.1016/j.ijpharm.2017.06.066.

(17) Potcoava, M. C.; Futia, G. L.; Gibson, E. A.; Schlaepfer, I. R. Lipid Profiling Using Raman and a Modified Support Vector Machine Algorithm. *J Raman Spectrosc* **2021**, *52* (11), 1910–1922. https://doi.org/10.1002/jrs.6238.

(18) Czamara, K.; Majzner, K.; Pacia, M. Z.; Kochan, K.; Kaczor, A.; Baranska, M. Raman Spectroscopy of Lipids: A Review. *J Raman Spectrosc* **2015**, *46* (1), 4–20. https://doi.org/10.1002/jrs.4607.

(19) Anantachaisilp, S.; Smith, S. M.; Treetong, A.; Pratontep, S.; Puttipipatkhachorn, S.; Ruktanonchai, U. R. Chemical and Structural Investigation of Lipid Nanoparticles: Drug–Lipid Interaction and Molecular Distribution. *Nanotechnology* **2010**, *21* (12), 125102. https://doi.org/10.1088/0957-4484/21/12/125102.

(20) Da Silva, E.; Bresson, S.; Rousseau, D. Characterization of the Three Major Polymorphic Forms and Liquid State of Tristearin by Raman Spectroscopy. *Chem Phys Lipids* **2009**, *157* (2), 113–119. https://doi.org/10.1016/j.chemphyslip.2008.11.002.

(21) Humphrey, W.; Dalke, A.; Schulten, K. VMD: Visual Molecular Dynamics. *J Mol Graph* **1996**, *14* (1), 33–38. https://doi.org/10.1016/0263-7855(96)00018-5.

(22) Benayad, Z.; von Bülow, S.; Stelzl, L. S.; Hummer, G. Simulation of FUS Protein Condensates with an Adapted Coarse-Grained Model. *J Chem Theory Comput* **2021**, *17* (1), 525–537. https://doi.org/10.1021/acs.jctc.0c01064.

(23) Virtanen, P.; Gommers, R.; Oliphant, T. E.; Haberland, M.; Reddy, T.; Cournapeau, D.; Burovski, E.; Peterson, P.; Weckesser, W.; Bright, J.; van der Walt, S. J.; Brett, M.; Wilson, J.; Millman, K. J.; Mayorov, N.; Nelson, A. R. J.; Jones, E.; Kern, R.; Larson, E.; Carey, C. J.; Polat, İ.; Feng, Y.; Moore, E. W.; VanderPlas, J.; Laxalde, D.; Perktold, J.; Cimrman, R.; Henriksen, I.; Quintero, E. A.; Harris, C. R.; Archibald, A. M.; Ribeiro, A. H.; Pedregosa, F.; van Mulbregt, P. SciPy 1.0: Fundamental Algorithms for Scientific Computing in Python. *Nat Methods* **2020**, *17* (3), 352–352. https://doi.org/10.1038/s41592-020-0772-5.

(24) Biological evaluation of medical devices — Part 5: Tests for in vitro cytotoxicity. *ISO 10993-5 Standard.*
